# Analyses of Plastome Sequences Improve Phylogenetic Resolution and Provide New Insight Into the Evolutionary History of Asian Sonerileae/Dissochaeteae

**DOI:** 10.3389/fpls.2019.01477

**Published:** 2019-11-21

**Authors:** Qiujie Zhou, Che-Wei Lin, Wei Lun Ng, Jinhong Dai, Tetsuo Denda, Renchao Zhou, Ying Liu

**Affiliations:** ^1^State Key Laboratory of Biocontrol and Guangdong Key Laboratory of Plant Resources, School of Life Sciences, Sun Yat-sen University, Guangzhou, China; ^2^Division of Botanical Garden, Herbarium of Taiwan Forestry Research Institute, Taipei, Taiwan; ^3^China-ASEAN College of Marine Sciences, Xiamen University Malaysia, Sepang, Malaysia; ^4^Laboratory of Ecology and Systematics, Faculty of Science, University of the Ryukyus, Nishihara, Japan

**Keywords:** Melastomataceae, Sonerileae, phylogenomics, biogeography, Phyllagathis, plastid genome

## Abstract

Sonerileae/Dissochaeteae (Melastomataceae) comprises ca. 50 genera, two thirds of which occur in Southeast Asia. Phylogenetic relationships within this clade remain largely unclear, which hampers our understanding of its origin, evolution, and biogeography. Here, we explored the use of chloroplast genomes in phylogenetic reconstruction of Sonerileae/Dissochaeteae, by sampling 138 species and 23 genera in this clade. A total of 151 complete plastid genomes were assembled for this study. Plastid genomic data provided better support for the backbone of the Sonerileae/Dissochaeteae phylogeny, and also for relationships among most closely related species, but failed to resolve the short internodes likely resulted from rapid radiation. Trees inferred from plastid genome and nrITS sequences were largely congruent regarding the major lineages of Sonerileae/Dissochaeteae. The present analyses recovered 15 major lineages well recognized in both nrITS and plastid phylogeny. Molecular dating and biogeographical analyses indicated a South American origin for Sonerileae/Dissochaeteae during late Eocene (stem age: 34.78 Mya). Two dispersal events from South America to the Old World were detected in late Eocene (33.96 Mya) and Mid Oligocene (28.33 Mya) respectively. The core Asian clade began to diversify around early Miocene in Indo-Burma and dispersed subsequently to Malesia and Sino-Japanese regions, possibly promoted by global temperature changes and East Asian monsoon activity. Our analyses supported previous hypothesis that *Medinilla* reached Madagascar by transoceanic dispersal in Miocene. In addition, generic limits of some genera concerned were discussed.

## Introduction

The Southeast Asia (SEA) is known for its complex geological history (Turner et al., 2001) and unique and rich biota (Mittermeier et al., 1999; Sodhi et al., 2004). This region, although covering only 4% of the earth’s land area, harbors approximately 20%–25% of the higher plant species on the planet (Woodruff, 2010; Corlett, 2014). According to Myers et al. (2000), SEA includes three out of the world’s eight hottest biodiversity hotspots for conservation priorities. However, how these diverse plants originate, disperse, and evolve remains largely unanswered. Phylogenetic and integrated systematic research, often even the basic taxonomic work (descriptions and revisions) which serve as the basis of conservation, are far from sufficient for many groups of organisms in SEA (Sodhi and Liow, 2000; Jin et al., 2018). The pantropical Sonerileae/Dissochaeteae complex, with its distribution centered in SEA, is one of such groups. Sonerileae (Melastomataceae) was originally circumscribed as including mainly paleotropical herbaceous species (Triana, 1865, Triana, 1871). Most authors followed Triana’s classification (Cogniaux, 1891; Krasser, 1893; Diels, 1932; Li, 1944; Chen, 1984a), but Van Vliet et al. (1981) and Renner (1993) proposed a wider circumscription of Sonerileae to include also the paleotropical Oxysporeae (shrubby) and the neotropical Bertolonieae (herbaceous). Recent molecular phylogenetic studies revealed that Sonerileae and Oxysporeae were nested with Dissochaeteae forming a well-supported Sonerileae/Dissochaeteae clade that showed no close relationship with core Bertolonieae (Clausing, 1999; Clausing et al., 2000; Clausing and Renner, 2001a; Clausing and Renner, 2001b; Renner et al., 2001; Fritsch et al., 2004; Goldenberg et al., 2012; Michelangeli et al., 2014; Veranso-Libalah et al., 2017; Veranso-Libalah et al., 2018; Bacci et al., 2019; Zhou et al., 2019). Sonerileae/Dissochaeteae comprises over 1000 species in ca. 50 genera, two thirds of which occur in SEA.

Generic delimitation and phylogenetic relationships within Sonerileae/Dissochaeteae are poorly understood, and many of the genera are of doubtful taxonomic validity, especially for those endemics of SEA (Cellinese, 1997; Clausing and Renner, 2001a). One prominent example would be *Phyllagathis* Blume, which contains approximately 70 species distributed from southern China, Indo-Burma to Sundaland (Cellinese, 2002; Chen and Renner, 2007; Wangwasit et al., 2010; Lin et al., 2015; Mathew et al., 2016; Tian et al., 2016; Lin et al., 2017; Vu et al., 2017). Transfers of species have been made between *Phyllagathis* and various genera, viz. *Anerincleistus* Korth. (Hansen, 1982; Maxwell, 1982; Cellinese, 2002), *Bredia* Blume (Li, 1944; Chen, 1984b), *Cyphotheca* Diels (Hu, 1952), *Plagiopetalum* Rehder (Chen, 1984b), *Scorpiothyrsus* H.L. Li (Li, 1944), and *Stapfiophyton* H.L. Li (= *Fordiophyton* Stapf) (Chen, 1984b). Publications of some small genera morphologically related to *Phyllagathis* had made the generic boundaries even more obscure (Chen, 1979; Chen, 1984b; Hansen, 1990; Hansen, 1992; Renner, 1993; Cellinese, 2002; Cellinese, 2003). Renner et al. (2001) and Clausing and Renner (2001a) represented the first comprehensive molecular studies of Sonerileae/Dissochaeteae, within which 29 species from 18 genera of this clade were sampled. Phylogenetic analyses confirmed the nesting of Sonerileae s.l. (including Oxysporeae) within Dissochaeteae, however, limited sampling of species prevent any discussion of generic limit in this clade. Zeng et al. (2016a,2016b), Zhou et al. (2018), Veranso-Libalah et al. (2018), and Bacci et al. (2019) focused mainly on *Fordiophyton*, *Bredia*, Melastomateae, and Bertolonieae respectively, sampling even fewer genera of Sonerileae/Dissochaeteae. Zhou et al. (2019) analyzed 109 species from 19 genera. This study clearly demonstrated the chaotic generic delimitation and succeeded in identifying a dozen of major lineages within Sonerileae/Dissochaeteae. However, the backbone phylogeny of this complex remained largely unresolved in all the above studies, severely hindering taxonomic revisions.

The estimated age and biogeographical history of Sonerileae/Dissochaeteae also remain controversial. Several studies have explored these issues using fossil-calibrated phylogeny based on DNA sequences of one or a few regions (*ndhF*, *rbcL*, *rpl16*, *accD-psaI*, *psbK-psbL*, nrITS, ETS, 18S, 26S) (Renner et al., 2001; Morley and Dick, 2003; Renner, 2004a; Renner, 2004b; Berger et al., 2016; Veranso-Libalah et al., 2018). The stem age of Sonerileae/Dissochaeteae was estimated to be 19 Mya (Renner et al., 2001), 38 Mya (Berger et al., 2016), 39.63 Mya (Veranso-Libalah et al., 2018), or 73 Mya (Morley and Dick, 2003). Previous authors had proposed three competing hypotheses on the biogeographical history of Sonerileae/Dissochaeteae: (1) diversification in SEA during Miocene and subsequent trans-oceanic dispersal of two sublineages from SEA to Madagascar and Africa; (2) dispersal from South America to Africa ca. 74 Mya ago followed by diversification and rafting on the Indian Plate to SEA (“Indian Ark” hypothesis); and (3) trans-Atlantic dispersal from South America to Africa during Late Eocene. The merit of these hypotheses needs further testing.

Previous studies were based on sequence data of nuclear ribosomal ITS (nrITS) and/or a few chloroplast markers (*trnV-trnM*, *ndhF*, *rbcL*, *rpl16*), both of which have their own limitations. nrITS, although proven useful in phylogenetics of Melastomateae (Michelangeli et al., 2012; Veranso‐Libalah et al., 2017; Veranso‐Libalah et al., 2018), failed to resolve the backbone of Sonerileae/Dissochaeteae. Chloroplast DNA sequences have been extensively used in phylogenetic analyses of angiosperms because of their conserved structure, high copy numbers, and uniparental inheritance (Birky, 1995). However, the use of only a few chloroplast genes is often insufficient to resolve genera or species level relationships due to low mutation rate (Shaw et al., 2007; Dong et al., 2012; Shaw et al., 2014). The advent of next-generation sequencing technologies offers a cost-effective means to obtain chloroplast genomic data, which have been successfully used to tackle difficult phylogenetic questions of plants from deep to shallow taxonomic level (Ma et al., 2014; Stull et al., 2015; Zhang et al., 2016; Heckenhauer et al., 2018; Niu et al., 2018; Wen et al., 2018). A chloroplast phylogenomic approach may help to better elucidate the relationships and biogeography of Sonerileae/Dissochaeteae.

This paper aims to (1) reconstruct the phylogenetic relationships in Sonerileae/Dissochaeteae, (2) infer the divergence times and biogeographical history, and (3) reassess the current generic delimitations based on the resulted phylogeny. To this end, we include in this study 138 species representing 23 genera in Sonerileae/Dissochaeteae, with a special emphasis on the widely distributed *Phyllagathis*.

## Materials and Methods

### Taxon Sampling

For phylogenomic analyses, 171 plastid genomes (152 species and five varieties) were included, representing one genus from Myrtaceae, and 42 genera from major lineages of Melastomataceae [Sonerileae/Dissochaeteae (23), Astronieae (1), Bertolonieae (1), Blakeeae (1), Cyphostyleae (1), Henrietteeae (1), Kibessieae (1), Marcetieae (1), Melastomateae (3), Memecyleae (1), Merianieae (2), Merianthera Group (1), Miconieae (2), Microlicieae (1), Rhexieae (1), Trioieneae (1)]. Twenty plastid genomes were obtained from previous reports (Reginato et al., 2016; Ng et al., 2017; Zhou et al., 2017; Tan et al., 2019), while the remaining were newly sequenced. For the highly polyphyletic *Phyllagathis*, 42 species were sampled from China, Vietnam, Malaysian Peninsula, and Borneo to cover its morphological and geographical range. A list of the taxa sampled in chloroplast phylogenomic analyses, their sampling locations, voucher information, and GenBank accession numbers were provided in **Table S1**.

Two phylogenomic datasets were assembled. (1) The Melastomataceae dataset, which contained all chloroplast genomes available in the family, was assembled for phylogenomic analysis. The resulted tree was also used as input tree in divergence time estimation and ancestral range reconstruction. The dataset was pre-analyzed using an outgroup from Myrtaceae (*Eucalyptus grandis* W. Mill ex Maiden). The most basal clade of Melastomataceae, *Memecylon-Pternandra*, was then selected as outgroup for this dataset. (2) The Sonerileae/Dissochaeteae dataset, which was used for comparison of phylogenies generated by chloroplast genome and nrITS sequence data. It comprised the chloroplast genomes of this clade and *Blakea schlimii* (Naudin) Triana (Blakeeae), with the latter selected as an outgroup.

To facilitate discussion, two additional datasets were assembled. An nrITS dataset parallel to the Sonerileae/Dissochaeteae genomic dataset (excluding *Opisthocentra clidemioides* Hook.f. as its nrITS sequence was not available) was analyzed for comparison. Another dataset of five concatenated plastid regions (*rbcL*, *rpl16*, *ndhF*, *psbK*-*psbL*, *accD*) (hereafter referred to as cp-5 gene dataset) with partially missing data was constructed to test the phylogenetic position of those African and South American species without available plastid genomic data. Please see **Table S2** for detailed sampling list and GenBank accession numbers for nrITS and plastid markers.

### DNA Isolation, Chloroplast Genome Sequencing

Total DNA was extracted from silica-gel dried leaves or fresh leaf tissue (when available) using the modified CTAB procedure (Doyle and Doyle, 1987) or using HiPure Plant DNA Mini Kit (Magen, Guangzhou, China) following the manufacturer’s protocols. Libraries were prepared from the total genomic DNA of 151 samples using Next Ultra II DNA Library Construction Kit (NEB, Beijing, China) following the manufacturer’s protocols. Shotgun sequencing was then performed on an Illumina HiSeq^™^ 2500 platform (150 bp paired-end reads) at Vazyme (Nanjing, China)/Novogene (Beijing, China).

The nrITS region was amplified and sequenced using universal primers (White et al., 1990) or assembled and extracted from our genomic shotgun sequencing reads. For polymerase chain reaction (PCR) amplification and sequencing, we followed the same procedure described in Zou et al. (2017). A mapping-based method was used to extract nrITS sequences from NGS sequencing data. First, nrITS sequences of most closely related species were applied as references to construct a BWA index (Li and Durbin, 2010). Short reads were then mapped to the reference with BWA-MEM. The resulting aligned SAM file were sorted and converted to BAM format. Single nucleotide polymorphisms (SNPs) and indels calling were conducted by SAMtools mplieup (Li et al., 2009) and BCFtools (https://github.com/samtools/bcftools). Finally, BCFtools was used to replace corresponding positions of reference with SNP information using *consensus* option, resulting in a FASTA sequence for a synthetic sequence of nrITS. Eighty nrITS sequences were newly generated.

### Plastid Genome Assembly and Annotation

To assemble the chloroplast genome of 151 samples, the total sequencing output, approximately 13 Gb of paired-end (PE z= 150 bp) sequence data per sample, was used as input into NOVOPlasty v1.2.4 (Dierckxsens et al., 2017). The partial sequence of *rbcL* (ribulose-1,5-bisphosphate carboxylase/oxygenase large subunit) of *Melastoma candidum* D. Don (GenBank accession number GQ436728) was adopted as the seed sequence in the seed-and-extend algorithm implemented in NOVOPlasty v1.2.4 (Dierckxsens et al., 2017). Annotation of the chloroplast genome was performed using the DOGMA online tool (Wyman et al., 2004) and then manually checked with the start/stop codons and junctions between introns and exons. The circular chloroplast genome maps were drawn with OGDRAW v1.3 (Lohse et al., 2007).

### Sequence Alignment

Chloroplast genome sequences were aligned using MAFFT v7.042 (Katoh and Standley, 2013) with default settings. Only one copy of the IRs was used in the final alignment to avoid overrepresentation of duplicated sequences. Dubiously aligned regions may bias phylogenetic inferences (Misof and Misof, 2009) and previous phylogenetic analyses of Melastomataceae based on plastid genome showed that among all the analytical schemes explored, only the non-coding regions without filtering of ambiguous aligned base pairs resulted in conflicting topology (Reginato et al., 2016). Therefore, we removed the poorly aligned regions from all phylogenomic datasets before subsequent analyses using trimAl v1.2 with “-gappyout” mode (Capella-Gutiérrez et al., 2009). Sequences of nrITS and the five plastid markers were aligned using SeqMan v7.1.0 (DNASTAR, Madison, WI, USA) and manually adjusted.

### Phylogenetic Analyses Based on Homogeneous Models

#### Data Partitioning

We explored the issue of data partitioning using the Melastomataceae phylogenomic dataset. Five partitioning schemes were employed in the maximum likelihood (ML) and Bayesian analyses: (1) no partitions, (2) two partitions, coding and noncoding sequences, (3) three partitions corresponding to large single copy (LSC) region, small single copy (SSC) region, and inverted repeat region (IR), (4) six partitions, *viz.* protein coding genes divided by three codon positions, tRNAs, rRNAs, and noncoding sequences, and (5) 15 partitions, which was determined as the best-fit partition scheme by PartitionFinder v2.1.1 (Lanfear et al., 2012), based on the following strategy. Firstly all protein coding genes were divided into 12 clusters by function as shown in **Figure S1** (each cluster was colored uniquely). For two of these clusters, “other genes” (*accD*, *ccsA*, and *cemA*) and “hypothetical chloroplast reading frame” (*ycf1*, *ycf2*, *ycf3*, and *ycf4*), which comprised several genes with different or unknown functions, each gene was further treated as a separate cluster. Each of the above 17 gene clusters was then divided into three subsets by codon position. With noncoding sequences, rRNA genes and tRNA genes treated as another three subsets, the Melastomataceae phylogenomic dataset was splitted into 54 subsets in total and then these subsets were assigned as input into PartitionFinder to select best-fit partitioning scheme and corresponding nucleotide substitution models.

#### Model Selection

The best-fitting models for each partition in the first four partitioning schemes as well as for nrITS dataset and cp-5 gene dataset were determined using the Akaike information criterion (AIC) (Posada and Buckley, 2004) in Modeltest version 3.7 (Posada and Crandall, 1998). For the fifth partitioning scheme, model for each partition is determined using PartitionFinder. For a summary of the model selection, see **Table S3**.

#### Bayesian Inference Analyses

Bayesian inference (BI) analyses were carried out in MrBayes 3.2.6 (Huelsenbeck and Ronquist, 2001) on the CIPRES cluster (Miller et al., 2010). When the model selected by Modeltest was not available in MrBayes, a more parameterized model was used (TVM was replaced by GTR, **Table 2** and **Table S3**). A recent empirical study has demonstrated that in certain situations, using both parameters I and G to accommodate rate variation across sites could lead to non-optimal values for both parameters (Moyle et al., 2012). Therefore, we also ran a parallel analysis replacing the selected model GTR+I+G with GTR+G and compared the results to detect potential parameter interaction. Two independent Markov chain Monte Carlo (MCMC) analyses were run each with four simultaneous chains (three heated and one cold) for 3,000,000 generations with the temperature parameter set to 0.08. Trees were sampled every 100 generations, with the first 7,500 trees (25%) discarded as burn-in, and the remaining trees were used to construct a 50% majority-rule consensus tree with Bayesian posterior probabilities (PP). Convergence was considered reached when the average standard deviation of split frequencies fell below 0.01. The effective sample sizes (ESS) were also assessed for all parameters and statistics using Tracer v1.7.1 (Rambaut et al., 2018). All ESS were obtained with values higher than 200, indicating that all parameters were sampled sufficiently for all chains to converge.

#### Maximum Likelihood Analyses

Maximum likelihood analyses were conducted in RAxML version 8.2.10 (Stamatakis, 2014) using the GTR+G model as recommended by the author. Node support was estimated with 1000 bootstrap replicates using a fast bootstrapping algorithm in RAxML (Stamatakis et al., 2008).

#### Comparisons of Partitioning Strategies

To compare the five partitioning strategies employed, we calculated marginal likelihood and Bayes factor (the ratio of marginal likelihoods from two competing models) using Tracer v1.7.1 (Rambaut et al., 2018) as described in Ma et al. (2014). We also used PartitionFinder to choose the best partition scheme by constraining the substitution model to GTR+G/GTR+I+G. The partition scheme with lowest AIC was considered to be the most fitting one. The best partition scheme was then applied to the final analyses of the two plastid phylogenomic datasets.

### Divergence Time Estimation

#### Dating Priors

Three dating priors were utilized, one secondary calibration from a recent study of Myrtales (Berger et al., 2016), and two fossils of Melastomataceae widely used in previous biogeographical studies of the family (Renner and Meyer, 2001; Morley and Dick, 2003; Renner, 2004a; Renner, 2004b; Veranso‐Libalah et al., 2018). The secondary calibration from Berger et al. (2016) is used to constrain the age of Melastomataceae *s.l.* (including *Memecylon*, node a) at 64.5 Ma [74.8–56.1 Ma, 95% highest posterior density (HPD)]. An Eocene fossil leaf from North America (Hickey, 1977) had the basic venation of Melastomataceae *s.s.* (excluding *Memecylon*). Conservatively, we used it to constrain the age of node b (excluding the most basal clade *Memecylon-Pternandra*) at 53 Ma. Another fossil prior is Miocene seed characteristic of Melastomateae and Rhexieae (Collinson and Pingen, 1992; Renner and Meyer, 2001). It was used to constrain the Melastomateae-Marcetieae-Rhexieae node (node c) at 26–23 Ma.

#### Beast Analyses

Divergence time estimation was performed in BEAST 2.5.2 (Bouckaert et al., 2014), using an uncorrelated lognormal relaxed clock with a birth-death speciation process (Kendall, 1948; Nee et al., 1994; Gernhard, 2008). Due to limited computational budget, sequences of nine protein coding genes (*atpB*, *matK*, *ndhF*, *psaB*, *psbB*, *rbcL*, *rpl2*, *rpoC2*, *rps4*; aligned length: 15984 bp) from nine gene clusters were extracted from the Melastomataceae dataset and assembled into a combined matrix as input alignment to BEAST. For the secondary calibration, we used a normal distribution with a standard deviation equivalent to the 95% HPD estimate of Berger et al. (2016). For the two fossil priors, a lognormal distribution with a mean of 1.5 and a standard deviation of 1 was adopted to allow for the possibility that the nodes are older than the fossils themselves (Sauquet, 2013; Berger et al., 2016). We ran two independent MCMC analyses, each of 350,000,000 generations sampling every 1,000 generations. The effective sampling of all parameters and convergence of independent chains were examined using Tracer version 1.7.1 (Rambaut et al., 2018). The obtained parameters and distribution of effective priors were broadly similar to those of corresponding specified priors, indicating that the calibration strategy was relatively reliable (**Figure S2**). LogCombiner v.2.4.5 (Bouckaert et al., 2014) was then used to combine the output files of independent runs, after the removal of 10% of samples as burn-in. Finally, estimated divergence time information was annotated to a constrained ML tree generated by the Melastomataceae dataset under best partition scheme using TreeAnnotator v.2.4.5 (Bouckaert et al., 2014).

### Ancestral Range Estimation

Ancestral range estimation was carried out using RASP (Yu et al., 2015). We used the annotated tree generated from BEAST analysis as input of the ancestral range estimation (ARE). The best-fit model Bayarealike+j was selected from the six models implemented in the software based on the AIC and likelihood ratio test results. We identified five geographical areas modified from Good (1974), Myers et al. (2000), and recent studies (Berger et al., 2016; Veranso‐Libalah et al., 2018): (A) North America; (B) South America; (C) Indo-Burma, also including part of southern and western Yunnan, southernmost Guangxi and Guangdong, and Hainan Island; (D) Sundaland; and (E) Sino-Japanese region, including most of central and southern mainland China, Taiwan, and Ryukyu. All the species in the dataset were coded as present or absent for each of the five areas (**Table S4**) based on herbarium specimens, literature (Chen, 1984a; Chen, 1984b; Hansen, 1985; Maxwell, 1989; Regalado, 1990; Cellinese, 2002; Cellinese, 2003; Chen and Renner, 2007; Kartonegoro and Veldkamp, 2010; Lin et al., 2015; Lin et al., 2017), and online database (Global Biodiversity Information Facility, http://www.gbif.org). No dispersal scenario and ancestral areas were assumed *a priori* for the analysis. In RASP we allowed the inferred ancestor to occupy up to two areas, corresponding to the maximum number of areas occupied by any extant species.

## Results

### Characteristics of Chloroplast Genomes

One hundred and fifty-one complete chloroplast genomes in Melastomataceae were newly sequenced and assembled in this study (**Table S1**). All newly obtained genomes are evolutionally conservative and similar to the ones previously published in Melastomataceae (Reginato et al., 2016; Ng et al., 2017; Zhou et al., 2017, Tan et al., 2019). Their genome sizes range from 153,291 to 158,960 bp with an average length of 155,986 bp. A total of 129 genes were annotated, *viz.* 84 protein-coding genes, 37 tRNA, and 8 rRNA, in all chloroplast genomes except that the *rpoC1* gene of *Sonerila cantonensis* Stapf (Liu 510) is pseudogenized. A gene map for the chloroplast genome of *Phyllagathis rotundifolia* (Jack) Blume is shown in **Figure S1** as a representative.

### Comparison of Different Partitioning Schemes

For each of the five partitioning schemes, trees generated by ML and BI analyses were broadly similar irrespective of the model used (GTR+G and GTR+I+G). Generally, BI analyses under selected GTR+I+G model recovered higher supported relationships than under GTR+G model (data not shown). Also, partition3 with best fit GTR+I+G model was favored over other partition schemes in both comparisons of AIC value and Bayes Factors (**Table 1**). Therefore, we applied partition3 to the final analyses of the two plastid phylogenomic datasets. For BI analyses, all three subsets (LSC, IR, and SSC) in partition3 scheme were analyzed with GTR+I+G (**Table 2**).

**Table 1:** Comparison of partitioning strategies used for the Melastomataceae dataset. The best partition scheme selected is indicated in bold.

Partitioning strategy	Description	Comparison with PartitionFinder				Comparison with BFs			
		GTR+G		GTR+G		GTR+G		GTR+I+G	
		ln L	AIC	ln L	AIC	Harmonic means (lnL)	2ln (BFs)^a^	Harmonic means(lnL)	2ln (BFs)
Partition1	All together	−859219	1719133	−857099	1714895	−859451.09	–	−82,8207.82	–
Partition2	Coding, noncoding	−886869	1774453	−884865	1770449	−877328.59	35755	−854485.52	52555.4
**Partition3**	**LSC, SSC, IRs**	−**848921**	**1698577**	−**847716**	**1696173**	−**849898.19**	−**19105.8**	−**818951.49**	−**18512.66**
Partition6	Noncoding, tRNAs, rRNAs,three codon positions	−881742	1764280	−877724	1760576	−886631.35	54360.52	−846168.71	35921.78
Partition15^b^	Identified by PartitionFinder	–	–	−877980	1761846	–	–	−837836.32	19257

a 2ln(BFs) equal to twice the difference of harmonic means (lnL) between Partition1 scheme and alternative partition schemes. We used the criterion of 2ln(BFs) of ≥10 as very strong evidence against the alternative schemes (Nylander et al., 2004).

b Under the substitution models selected by PartitionFinder.

**Table 2:** Data characteristics, substitution model selected and used in ML/BI analysis for the four datasets used in this study.

Dataset	Subset	Taxa	Number of sites	Missing data (%)	Variable sites/PIS*	Best fit model	ML	BI
Melastomataceae dataset	LSC	170	85,815	–	34474/20827	GTR+I+G	GTR+G	GTR+I+G
	IR		26,500	–	2482/977	GTR+I+G	GTR+G	GTR+I+G
	SSC		16,897	–	7878/5127	TVM+I+G	GTR+G	GTR+I+G
Sonerileae/ Dissochaeteae dataset	LSC	150	85,474	–	23183/13083	GTR+I+G	GTR+G	GTR+I+G
	IR		24,923	–	1258/501	GTR+I+G	GTR+G	GTR+I+G
	SSC		18,642	–	5680/3420	TVM+I+G	GTR+G	GTR+I+G
nrITS dataset	–	149	750	–	457/389	GTR+I+G	GTR+G	GTR+I+G
cp-5 gene dataset	–	188	4,022	12.02	1137/696	GTR+I+G	GTR+G	GTR+I+G

*Parsimoniously informative sites.

### Phylogenetic Analyses

**Figure 1:**
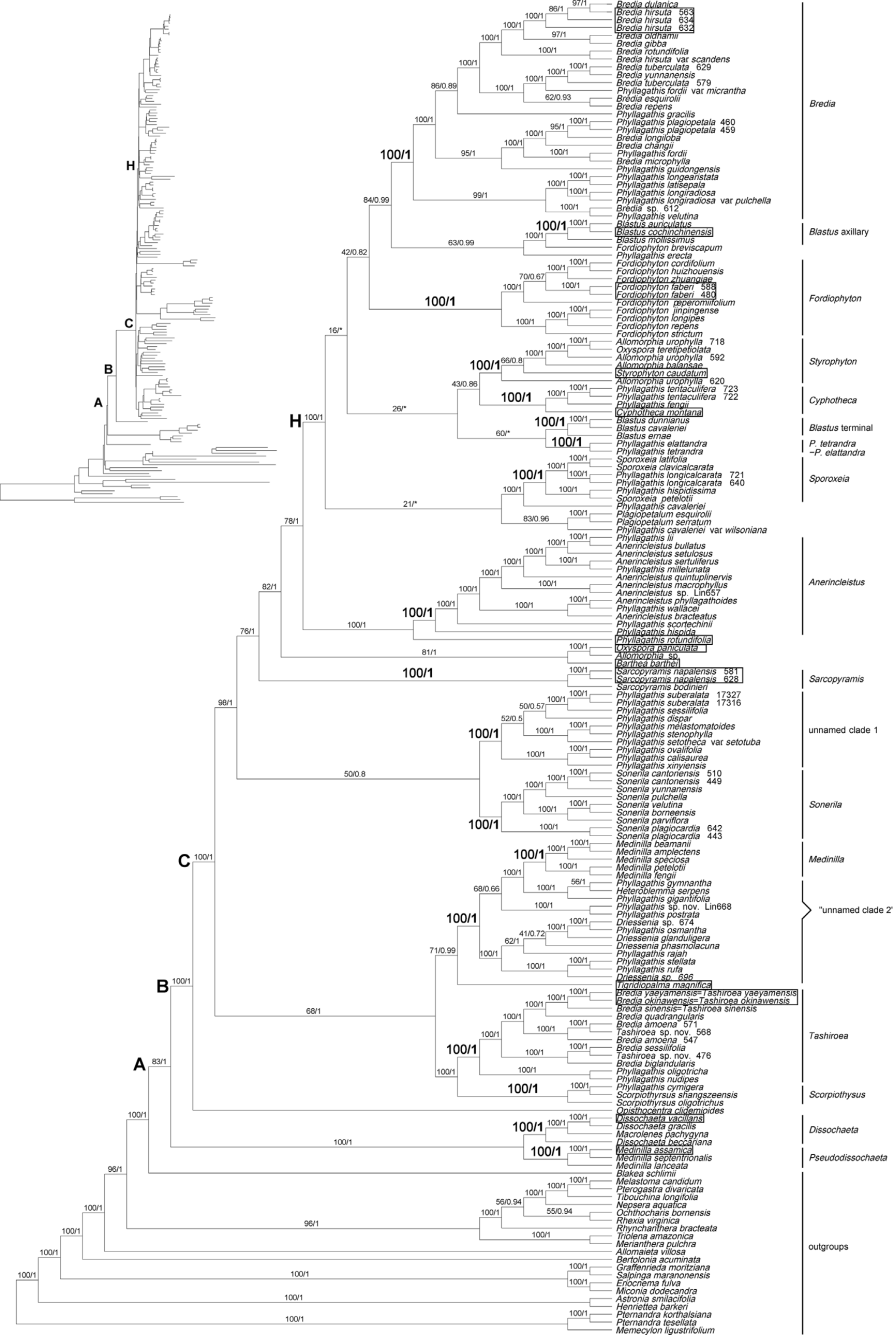
Maximum likelihood (ML) phylogenetic tree of Melastomataceae based on chloroplast genome sequences with 112 genes included shown as a cladogram (phylogram inset). Bootstrap values obtained from ML analyses (left) and Bayesian posterior probabilities resulting from Bayesian inference (BI) (right) are given on the branches. An asterisk denotes a branch collapsed in BI. The types of the genera sampled in Sonerileae/Dissochaeteae are indicated with boxes. Clades A, B, C, and H represent the nodes discussed in the text. “Unnamed clade 2” denotes a group of species which comprised the unnamed clade 2 in the nrITS tree (**Figure S4**).

Statistics of sequences sampled in Melastomataceae and Sonerileae/Dissochaeteae phylogenomic datasets are summarized in **Table 2**. Trees generated by BI and ML analyses had nearly identical topology, except that four/three nodes with weak support in ML analyses collapsed in BI analyses. The ML trees resulting from analyses of the two datasets are shown in **Figures 1** and **S3**, with Bayesian PP and ML bootstrap support values (BS) indicated on the branches. We considered a certain clade as resolved when BS was ≥70%, and PP was ≥0.99.

Analyses of the Melastomataceae dataset revealed that all genera sampled in Sonerileae/Dissochaeteae, except *Ochthocharis* Blume, formed a strongly supported clade (PP = 1.00, BS = 83%) (**Figure 1**). *Ochthocharis*, previously placed in Sonerileae, showed closer relationship with Rhexieae, Marcetieae, Melastomateae, and Microlicieae instead of other genera in Sonerileae/Dissochaeteae. Phylogenetic relationships within Sonerileae/Dissochaeteae inferred from the two chloroplast phylogenomic datasets were nearly identical (**Figures 1** and **S3**). The Asian *Dissochaeta-Pseudodissochaeta* (PP = 1.00, BS = 100%) was recovered as the most basal clade in the complex, followed by a split (PP = 1.00, BS = 100%) between the South American *Opisthocentra* Hook.f. and a large clade consisting of the remaining Asian species (PP = 1.00, BS = 100%) (**Figures 1** and **S2**). As shown in **Figure 1**, 17 species clusters were recovered in Sonerileae/Dissochaeteae. The backbone phylogenies of the complex were only partially supported. Relationships among major lineages in node H remained unresolved (**Figure 1**). The details of trees based on the nrITS and cp-5 gene datasets were shown in **Table 2** and **Figures S4** and **S5**.

### Divergence Time Estimates

**Figure 2:**
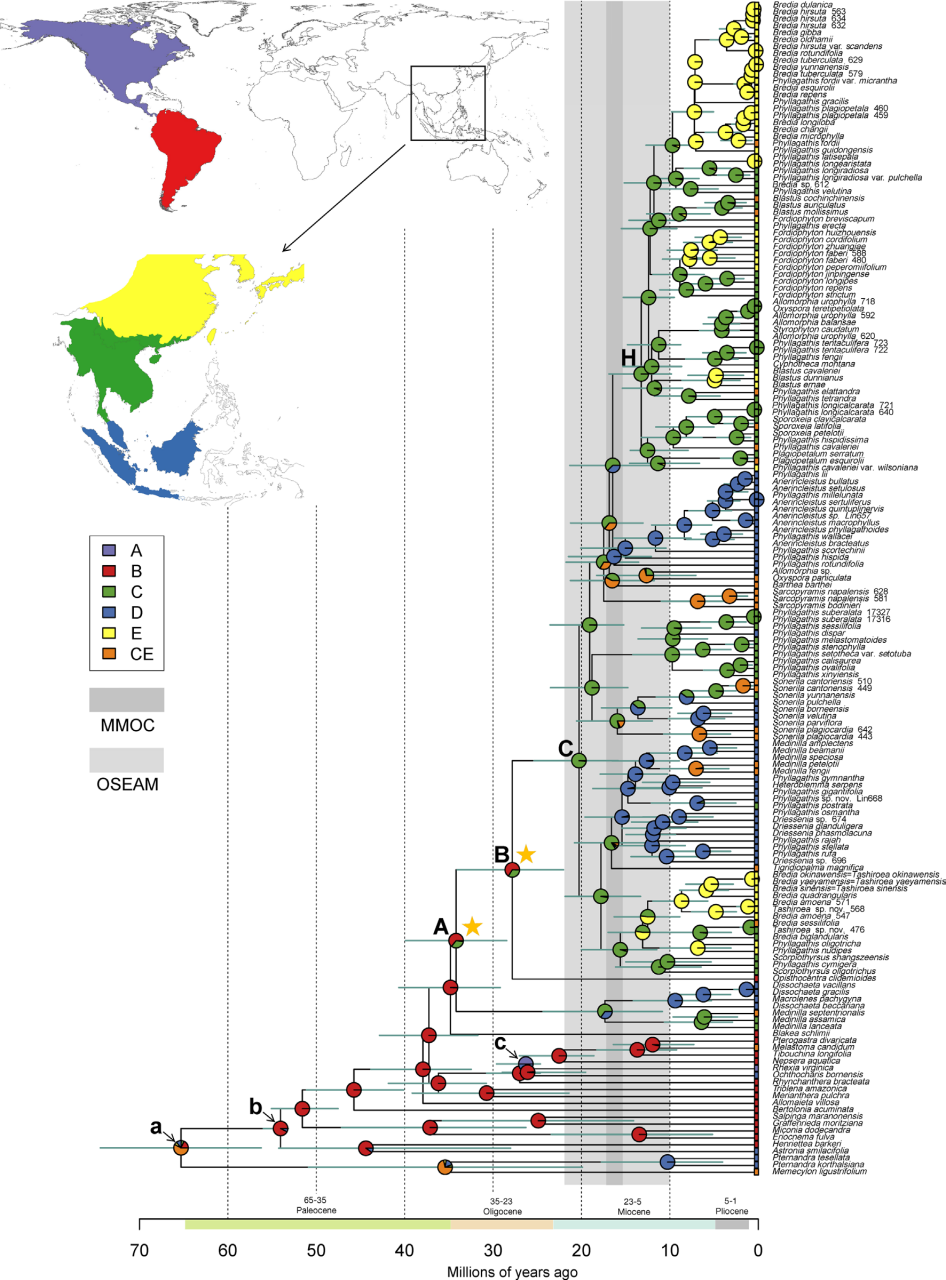
Constrained maximum likelihood phylogenetic tree of Melastomataceae annotated with estimated divergence time and ancestral range obtained from BEAST and RASP analyses (see *Materials and methods*). Mean divergence times are shown with their 95% highest posterior density (HPD: green bars). Clades A, B, C, and H represent nodes discussed in the text, whereas node a, b, and c indicate the calibration points. Pie charts represent the area probability inferred for each node. The inset map shows the coding of five biogeographical areas. The present-day distribution is given at the tip for each species. The light and darker vertical bars denote two climatic events: onset of East Asia monsoons and its strengthening to maximum in Miocene (OSEAM, 10–22 Mya) and the Mid Miocene Climate Optimum (MMOC, 15.5–17.2 Mya), respectively. Stars denote two dispersal events (node A, B) of Sonerileae/Dissochaeteae from South America to the Old World.

The dated phylogeny obtained from the BEAST analysis is shown in **Figure 2**. Divergence time estimates suggested that the Sonerileae/Dissochaeteae complex originated during late Eocene (stem age: 34.78 Mya; 95% HPD: 29.07–40.53 Mya). The most basal clade in the complex, *Dissochaeta-Pseudodissochaeta*, diverged around 33.96 Mya (late Eocene; 95% HPD: 28.33–39.8 Mya). The subsequent split of the South American *Opisthocentra* and Asian clades (node C) was estimated to be around 27.79 Mya (Mid Oligocene; 95% HPD: 21.91–33.94 Mya). The Asian clade (node C) began to diversify during early Miocene (crown age: 20.25 Mya; 95% HPD: 15.71–25.24 Mya). Divergence time estimations and respective 95% HPD intervals for nodes of interest were listed in **Table 3**.

**Table 3:** Stem and crown median age estimates and 95% highest posterior density (HPD) for Sonerileae/Dissochaeteae and other clades of interest based on BEAST analysis.

Clade name	Stem age (Mya)	Crown age (Mya)	Ancestral area (Bayarealike+j)
Node A, Sonerileae/Dissochaeteae	34.78 (29.07–40.53)	33.96 (28.33–39.8)	B (64.40),C (33.89)
Node B	33.96 (28.33–39.8)	27.79 (21.91–33.94)	B (66.02),C (33.86)
Node C	27.79 (21.91–33.94)	20.25 (15.71–25.24)	C (98.43)
Node H	16.44 (12.08–21.2)	13.22 (9.7–16.7)	C (99.83)
*Bredia*	11.8 (8.71–15.04)	9.7 (6.86–12.58)	C (94.50)
*Blastus* axillary	8.94 (5.34–12.5)	4.08 (1.71–6.75)	C (95.00)
*Fordiophyton*	12.22 (9.08–15.37)	8.87 (6–12.5)	C (97.11)
*Styrophyton*	11.25 (8.65–14.67)	4.11 (2.4–6.52)	C (100)
*Cyphotheca*	11.25 (8.65–14.67)	4.9 (2.16–8.08)	C (100)
*Blastus* terminal	11.74 (8.44–15.24)	4.93 (1.95–8.49)	E (100)
*P. tetrandra-* *P. elattandra*	11.74 (8.44–15.24)	7.84 (4.2–11.56)	C (97.62)
*Sporoxeia*	9.6 (6.26–13.1)	8.12 (4.9–11.46)	C (100)
*Anerincleistus*	16.31 (11.97–21.35)	15.02 (10.31–19.96)	D (100)
*Sarcopyramis*	17.16 (13.42–21.62)	6.84 (3.35–10.84)	CE (99.72)
Unnamed clade 1	18.78 (14.63–23.38)	9.7 (5.89–14.06)	C (100)
*Sonerila*	18.78 (14.63–23.38)	15.9 (11.87–20.43)	C (78.50),CE (18.26)
*Medinilla*	13.87 (10.13–17.76)	12.61 (8.81–16.4)	D (95.35)
Unnamed clade 2	16.46 (12.28–20.67)	15.38 (11.5–19.47)	D (96.39)
*Tashiroea*	15.58 (11.35–19.89)	13.05 (9.23–16.87)	C (46.99), E (53.01)
*Scorpiothysus*	15.58 (11.35–19.89)	11.23 (6.35–16.01)	C (100)
*Dissochaeta*	17.31 (10.72–24.22)	9.37 (5.19–14)	D (100)
*Pseudodissochaeta*	17.31 (10.72–24.22)	6.38 (2.79–10.49)	C (99.96)

Ancestral ranges and relative probabilities (p > 10%) of these clades estimated under the Bayarealike+j model are also shown. North America* (A);*South America* (B);*Indo-Burma* (C);*Sundaland* (D);*Sino-Japanese region* (E).*

### Biogeographical History

Ancestral range reconstruction for Melastomataceae using RASP is shown in **Figure 2**, with the area probability inferred for each node represented by pie charts. The ancestral areas of Sonerileae/Dissochaeteae (node A) and the core Asian Sonerileae/Dissochaeteae (node C) were estimated to be South America with moderate support (area B, p = 0.64; **Figure 2**, **Table 3**) and Indo-Burma with strong support (area C, p = 0.98; **Figure 2**, **Table 3**), respectively. Thirty-three dispersal events were detected within Sonerileae/Dissochaeteae, of which two were intercontinental dispersals from South America to Asia [33.96 Mya (95% HPD: 28.33–39.8 Mya), node A; 27.79 Mya (95% HPD: 21.91–33.94 Mya), node B] and the remaining were dispersals among different regions of SEA. The most common dispersals in SEA were those from Indo-Burma to Sino-Japanese region (19 out of 31) and from Indo-Burma to Sundaland (5 out of 31). Age estimation showed ongoing dispersals from Indo-Burma to Sino-Japanese region during the past 20 Mya (19.64–0.87 Mya), whereas those from Indo-Burma to Sundaland were relatively ancient, ranging from 17.31 to 9.46 Mya. Ancestral ranges and relative probabilities of clades of interest are given in **Table 3**.

## Discussion

### Comparison of Trees Generated by Chloroplast Genome and nrITS Sequence Data

**Figure 3:**
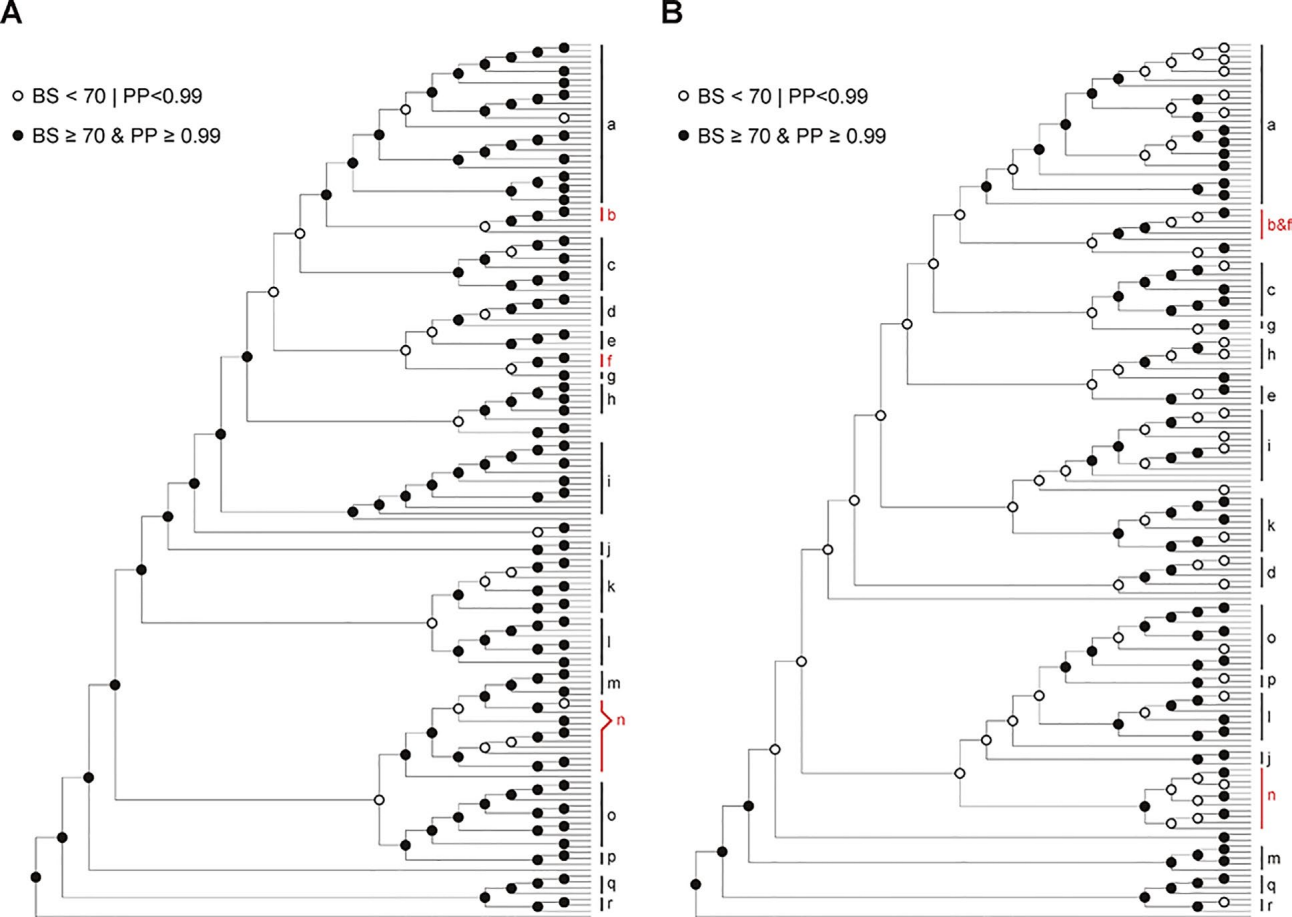
Comparison of trees based on chloroplast genomic dataset **(A)** and nrITS dataset **(B)**. Solid circles and circles denote nodes with strong and weak support (BS ≥ 70 and PP ≥ 0.99 vs. BS < 70 or PP < 0.99) respectively. Clades strongly supported in both trees are indicated with black bars (a, *Bredia*; c, *Fordiophyton*; d, *Styrophyton*; e, *Cyphotheca*; g, *P. tetrandra−P. elattandra*; h, *Sporoxeia*; i, *Anerincleistus*; j, *Sarcopyramis*; k, unnamed clade 1; l, *Sonerila*; m, *Medinilla*; o, *Tashiroea*; p, *Scorpiothysus*; q, *Dissochaeta*; r, *Pseudodissochaeta*.), whereas those supported in only one tree are marked with red bars (b, *Blastus* axillary; f, *Blastus* terminal; b & f, *Blastus*; n, unnamed clade 2). Also see **Figures S3**–**S4**.

The phylogenetic tree generated from chloroplast genomic data is generally better resolved than the nrITS tree in terms of relationships both within and among major clades (**Figures 1**, **S3**, and **S4**). As shown in **Figure 3**, 87% of the nodes in the plastid tree received moderate to strong support comparing to 55% in the nrITS tree. The phylogenetic affiliation of several species, unresolved in the nrITS phylogeny, were recovered by plastid phylogenomic data. For example, *Phyllagathis rotundifolia*, the type of *Phyllagathis*, was recovered as the sister group of *Anerincleistus* clade (**Figures 1** and **S3**). Nevertheless, plastid phylogenomic analyses failed to fully resolve the backbone phylogeny of Sonerileae/Dissochaeteae (**Figures 1** and **S3**). The weakly supported short internodes following node C and node H, together with our divergence time estimations, indicate putative rapid radiation around early (20.25 Mya) and middle Miocene (13.22 Mya). Therefore, even chloroplast genomic sequences cannot satisfactorily resolve the relationships among clades of Sonerileae/Dissochaeteae that evolved through rapid radiation.

Comparison of the plastid and nrITS trees revealed several strongly supported incongruences regarding the interspecific relationships within some species clusters, e.g. *Anerincleistus* clade, *Bredia* clade, *Medinilla* clade, and *Tashiroea* clade. The factors commonly invoked as the potential causes of incongruence between plastid and nrITS phylogenies include sampling error, long-branch attraction, incomplete lineage sorting, hybridization, and subsequent introgression (Rieseberg and Soltis, 1991; Soltis and Kuzoff, 1995; Soltis et al., 1996; Wendel and Doyle, 1998). Sampling error can be ruled out as the main factor based on the high PP and BS values of the incongruent topologies (PP = 1.00, BS > 80%). The possibility of long-branch attraction (LBA) is also rejected for two reasons. The data were analyzed using model-based methods that are less sensitive to LBA, besides, no long terminal branches were involved in these incongruences. Ancestral polymorphism may survive recent speciation leading to discordant gene trees at interspecific levels (Wendel and Doyle, 1998; Knowles and Carstens, 2007). Hybridization and the transfer of alleles across the species barrier is also widespread in plants, especially for closely related species. Therefore, both lineage sorting and hybridization could be the cause for interspecific level incongruence.

The plastid genome tree and nrITS tree are congruent in most major lineages of Sonerileae/Dissochaeteae. Of the 17 major clades recognized in the nrITS tree, 15 were also strongly supported by plastid genomic data (**Figures 1**, **S3**, and **S4**). Incongruences lies in two clades, *viz.* the *Blastus* clade and unnamed clade 2. *Blastus* Lour. is morphologically highly homogeneous and distinct from other genera. This genus, although recovered as monophyletic in the nrITS phylogeny (**Figure S4**), formed two separate clades in the plastid genome tree corresponding to axillary and terminal inflorescences (**Figures 1** and **S3**). The unnamed clade 2 contains species sampled in *Driessenia* Korth, *Phyllagathis*, and *Heteroblemma* (Blume) Cámara-Leret, Ridd.-Num. & Veldkamp. It was well recognized in nrITS phylogeny (**Figure S4**) but was revealed to be paraphyletic in the plastid phylogeny (**Figures 1** and **S3**), forming a larger lineage together with the *Medinilla* clade and two species of *Phyllagathis* from Vietnam, *P. prostrata* C. Hansen, and an undescribed new species. However, detection of strongly supported discordance and assessment of the potential causes such as hybridization and introgression are hampered by poor resolution of some parts of the phylogenetic trees.

Chloroplast phylogenomic data together with the even less informative nrITS sequence data failed to fully tackle the phylogenetic relationships within Sonerileae/Dissochaeteae. In future analyses, plastid genomic data should be combined with sequence data from multiple nuclear genes to unravel the phylogeny of Sonerileae/Dissochaeteae and the underlying evolutionary processes.

### Origin and Biogeography

Sonerileae/Dissochaeteae exhibits a disjunct distribution between South America and the Old World, with its distribution centered in SEA. Molecular dating and biogeographical analyses indicated a South American origin for this clade during late Eocene (stem age: 34.78 Mya; 95% HPD: 29.07–40.53 Mya; **Figure 2**, **Table 3**). Our result agrees with Berger et al. (2016) and Veranso-Libalah et al. (2018) who estimated the age of Sonerileae/Dissochaeteae to be 38 and 39.63 Mya respectively based on limited sampling of this clade. Two previous age estimations for this clade, 19 Mya (Renner et al., 2001) and 73 Mya (Morley and Dick, 2003), are not supported with our data.

At the base of Sonerileae/Dissochaeteae, an Asian clade *Dissochaeta-Pseudodissochaeta* (stem age: 33.96 Mya; 95% HPD: 28.33–39.8 Mya) branched off first followed by a split at node B (27.79 Mya; 95% HPD: 21.91–33.94 Mya) between the South American clade *Opisthocentra* and the remaining Asian species (node C) (**Figure 2**). Ancestral range estimation indicated two dispersal events from South America to the Old World during late Eocene (33.96 Mya; 95% HPD: 28.33–39.8 Mya) (node A) and Mid Oligocene (27.79 Mya; 95% HPD: 21.91–33.94 Mya) (node B) respectively. Phylogenetic analyses of the cp-5 gene dataset revealed that within node B the South American *Opisthocentra*, *Boyania* Wurdack, *Phainantha* Gleason and the African clade comprising *Gravesia* Naudin, *Calvoa* Hook.f., *Dicellandra* Hook.f., and *Amphiblemma* Naudin diverged successively, followed by a subsequent split of the Asian clade (node C) (**Figure S5**). The same pattern is also observed in a most recent phylogenetic study of Bertolonieae and Sonerileae/Dissochaeteae (Bacci et al., 2019). These data contradicted the previous view that *Gravesia*, *Calvoa,* and *Amphiblemma* arrived in Africa and Madagascar *via* long-distance dispersal from SEA (Clausing and Renner, 2001b; Renner et al., 2001; Renner, 2004a; Renner, 2004b), clearly indicating a second dispersal event from South America to Africa and Asia in node B. Based on the inferred age of the two dispersal events [33.96 Mya (95% HPD: 28.33–39.8 Mya) and 27.79 Mya (95% HPD, 21.91–33.94 Mya)], the “Indian Ark” hypothesis (Morley and Dick, 2003) is refuted. There are two alternatives. Direct trans-Atlantic dispersal of the lineages to the Old World is possible *via* oceanic steeping stones. Also, the basal lineages might have migrated from South America to North America and then entered Eurasia through the North Atlantic land bridge and spread to Africa and SEA during Eocene when the global temperature was still high. The latter hypothesis is supported by Eocene and Miocene Melastomataceae fossils discovered from North America and Europe (Hickey, 1977; Collinson and Pingen, 1992; Wehr and Hopkins, 1994). Both scenarios were proposed for Melastomateae (Veranso-Libalah et al., 2018), another tribe with transtropical disjunction in the family.

The core Asian Sonerileae/Dissochaeteae (node C) began to diversify around early Miocene (crown age: 20.25 Mya; 95% HPD: 15.71–25.24 Mya) in Indo-Burma and dispersed subsequently southward to Malesia and northward to Sino-Japanese (**Figure 2**). This result is congruent with the findings of previous meta-analyses, which showed that Indo-Burma’s biota (Indochina) had been predominantly characterized by *in situ* diversification and subsequent emigration since at least the early Miocene (De Bruyn et al., 2014). A series of short internodes with unsatisfactory resolution were observed following node C and node H, indicating the onset of rapid radiation around early (20.25 Mya, 95% HPD: 15.71–25.24 Mya) and middle Miocene (13.22 Mya, 95% HPD: 9.7–16.7 Mya) respectively (**Figures 1** and **2**). The early Miocene collision of Australia with the eastern margin of Sundaland (Hall, 2009), the onset of East Asia monsoons in early Miocene and its strengthening to maximum in Mid Miocene (Sun and Wang, 2005; Guo et al., 2008) and the Mid Miocene Climate Optimum (MMOC, 15.5–17.2 Mya) resulted in increased topographic complexity, wet and warm climate, and development of widespread evergreen rainforest. These factors might in turn promoted Miocene radiation of Asian Sonerileae/Dissochaeteae in Indo-Burma and their subsequent dispersal into Malesia and Sino-Japanese. The link between warmer and wetter climate and higher speciation was confirmed in a recent study (Kong et al., 2017), showing that global temperature changes and East Asian monsoons had played crucial roles in floristic diversification.

Finally, our analyses estimated a stem age of 13.87 Mya (95% HPD: 10.13–17.76 Mya) for the bird dispersed *Medinilla* clade (**Figure 2**). Analyses of the cp-5 gene dataset showed that the Madagascan species of *Medinilla* were nested within Asian species, branching off after *M. fengii-M. petelotii* (**Figure S5**). Therefore, we agree with Renner et al. (2001) and Renner (2004a) that *Medinilla* reached Madagascar by transoceanic dispersal in Miocene, possibly after 12.61 Mya (95% HPD: 8.82–16.4 Mya), the stem age of *M. fengii-M. petelotii* (**Figure 2**).

### Taxonomic Implications

Of the 23 genera sampled from Sonerileae/Dissochaeteae, only *Ochthocharis* fell out of this clade in the present analyses. As shown in **Figure 1**, it was close to Rhexieae, Marcetieae Melastomateae, and Microlicieae, conforming to the finding of Veranso-Libalah et al. (2018). *Ochthocharis* is a distinct genus with an African-Asian distribution, comprising nine species mostly found in the coastal lowland in wet and riverine habitats. Morphologically, it is readily distinguished by the indumentum, the structure of ovary, fruit, and seeds, showing no close resemblance to any other Asiatic genus (Hansen and Wickens, 1981). *Ochthocharis* should be excluded from Sonerileae/Dissochaeteae based on molecular data. However, a broader sampling of the genus and its close relatives (Rhexieae, Microlicieae, etc.) is still needed to resolve their phylogenetic relationships.

A dozen of major lineages in Sonerileae/Dissochaeteae, well recognized in both nrITS and plastid genomic phylogeny (**Figures 1**, **S3** and **S4**), are uncovered in this study, which provides some important insights for taxonomy. A comparison of these lineages is shown in **Table S5**.

#### Dissochaeta

*Dissochaeta* Blume is a genus of woody climbers occurring in SEA. Early authors had established a number of genera closely resembled *Dissochaeta*, *viz. Anplectrum* A.Gray, *Dalenia* Korth., *Diplectria* (Blume) Rchb., *Omphalopus* Naudin, *Backeria* Bakh.f., *Neodissochaeta* Bakh.f., and *Macrolenes* Naudin (Reichenbach, 1841; Korthals, 1844; Naudin, 1851; Gray, 1854; Bakhuizen, 1943). All these genera are woody climbers with a scrambling habit. Generic delimitations are mainly based on position of inflorescence, morphology of hypanthium, calyx lobes, and especially stamens (Kartonegoro et al., 2018). Previous molecular phylogenetic study (Clausing and Renner, 2001a) showed that *Macrolenes* was sister to *Diplectria*, and *Macrolenes*-*Diplectria* to *Dissochaeta*. Renner et al. (2001) thus synonymized *Diplectria* and *Macrolenes* under *Dissochaeta*. Kartonegoro et al. (2018), emphasizing that *Macrolenes* differs from *Dissochaeta* in the axillary inflorescence, the hair cushion domatia on the abaxial surface of leaf blade, long and persistent calyx lobes, and anthers with several basal filiform appendages, preferred to retain *Macrolenes* as a distinct genus. According to the present study, *Macrolenes pachygyna*, a species with the above character combination, is nested within species of *Dissochaeta*. Inclusion of *Macrolenes* in *Dissochaeta* is therefore supported.

#### Pseudodissochaeta

*Pseudodissochaeta* Nayar is a small genus of shrubs or small trees endemic to Indochina. Nayar (1969) proposed that this genus is close to *Dissochaeta*, but differs in the erect habit, connectives hardly produced and ventrally biauriculate, and extra-ovarial chambers descending to the middle or the base of the ovary. However, Chen (1983) considered *Pseudodissochaeta* as a congener of *Medinilla* and reduced the former. Chen (1984a) and Chen and Renner (2007) followed this treatment, while Renner et al. (2001) recognized *Pseudodissochaeta* as a distinct genus. Our phylogenetic analyses included three species previously treated in *Pseudodissochaeta*, *viz. M. assamica* (C. B. Clarke) C. Chen (the type of *Pseudodissochaeta*), *M. septentrionalis* (W. W. Sm.) H. L. Li, and *M. lanceata* (Nayar) C. Chen. *Pseudodissochaeta* was recovered as monophyletic and sister to *Dissochaeta*, while the rest species sampled in *Medinilla* formed a clade close to *Heteroblemma*, *Driessenia*, and some species of *Phyllagathis* (**Figures 1**, **S3** and **S4**). The generic status of *Pseudodissochaeta* should be retained.

#### Anerincleistus

The *Anerincleistus* clade comprises five species of *Phyllagathis* and eight species of *Anerincleistus*, including *A. macrophyllus* Bakh.f., a species morphologically close to *A. hirsutus* Korth., the type of this genus. Species of this clade occur in Borneo, Malay Peninsula, and Sumatra. They are similar in having eight isomorphic stamens with minute connective appendages, but are quite diverse in habit, leaf morphology, inflorescence morphology, and capsule morphology (**Table S5**). Analyses of nrITS sequence data failed to resolve the phylogenetic affiliation of this clade (**Figure S4**), but chloroplast phylogenomic analyses recovered it as sister to the type of *Phyllagathis* with strong support (**Figures 1** and **S3**). Based on this result, *Phyllagathis* should be recircumscribed to include this clade. Nevertheless, this relationship needs to be further tested using nuclear sequence data other than nrITS before formal taxonomic treatment.

#### Bredia *and* Tashiroea

Molecular phylogenetic analyses reveal that the type of *Bredia* is nested in a clade of 21 species, while *Tashiroea*, a genus previously synonymized in *Bredia*, falls in another distantly related clade of 10 species (**Figures 1**, **S3**, and **S4**). Both clades are distributed in southern mainland China, Taiwan, and the Ryukyu islands. Phylogenetically, the *Tashiroea* clade is sister to *Scorpiothysus* and the *Bredia* clade is nested in an internally unresolved larger branch with *Blastus*, *Fordiophyton*, etc. Morphologically, they differ in a series of characters including indumentum, texture of leaves, and capsule morphology (**Table S5**). Molecular and morphological evidence confirm that the *Bredia* and *Tashiroea* clades represent distantly related lineages. *Bredia* should be recircumscribed to include the former clade and *Tashiroea* should be resurrected for the latter.

#### Cyphotheca *and* Sporoxeia

The *Cyphotheca* clade comprises the monotypic *Cyphotheca*, *Phyllgathis fengii* C. Hansen, and *P. tentaculifera* C. Hansen. The *Sporoxeia* clade contains *Sporoxeia, P. hispidissima* (C. Chen) C. Chen, and *P. longicalcarata* C. Hansen. Both clades are nested in the internally poorly resolved node H with *Bredia* clade, *Fordiophyton* clade, *Blastus* and *Styrophyton* clade, etc. (**Figures 1**, **S3**, and **S4**). The two clades, *Cyphotheca* and *Sporoxeia*, are morphologically distinct from other clades within node H in the mature ovary crown enclosing an obpyramidal space and 4-horned placental column (**Table S5**). From each other, they differ in anther morphology (connectives thickened without dorsal appendage vs. connectives dorsally spurred). Although the phylogenetic relationships in node H needs to be further tested, both *Cyphotheca* and *Sporoxeia* may have to be expanded to include additional species currently placed in *Phyllagathis*.

#### Scorpiothysus

This clade includes the highly homogeneous *Scorpiothysus* (Yunnan, Guangxi, Hainan), *Phyllagathis cymigera* C. Chen (Yunnan) (this study), and also *P. hainanensis* (Merr. & Chun) C. Chen (Hainan) (Zhou et al., 2019). *Scorpiothysus* differs from *P. hainanensis* and *P. cymigera* in the scorpioid inflorescence, but they share general resemblance in leaf, stamen, and capsule morphology (**Table S5**) some of which were long noticed by Hansen (1992). Phylogenetic analyses consistently placed the *Scorpiothysus* clade near the *Tashiroea* clade with strong support (**Figures 1**, **S3** and **S4**). Morphologically, it differs from the latter clade in 7–9-veined leaves, often scorpioid cymose panicles and anther with 2-setose ventral appendages and is better treated as a separate genus. *Scorpiothysus* should be expanded to include *P. hainanensis* and *P. cymigera*.

#### Unnamed Clade 1

This clade consists of ten species of *Phyllagathis* occurring in southernmost mainland China (6 spp), Vietnam (2 spp), and Borneo (2 spp) (Zhou et al., 2019; this study). The close relationship among some of these allopatric species in *Phyllagathis*, proposed by Hansen (1992), is confirmed here. Species in this clade are similar in the isomorphic stamens, dorsally spurred connectives, terminal inflorescences, umbellate or cymose (rarely terminal or axillary solitary flower), enlarged ovary crown forming an obpyramidal depression on the top, horned placental column, and thready placenta. The phylogenetic affiliation of unnamed clade 1 is poorly resolved. But this clade showed no close relationship with the type of *Phyllagathis* in all analyses. If these results reflect the true relationships, species in this clade should be excluded from *Phyllagathis* and treated as a distinct genus.

## Data Availability Statement

All the sequencing data generated in this study has been deposited in GenBank with accession numbers MK994778-MK994928 (complete chloroplast genome sequences) and MN031159-MN031238 (ITS sequences).

## Author Contributions

RZ and YL designed the experiments. All authors took part in the field work. QZ and JD carried out the experiment. QZ, RZ, and YL analyzed the data. QZ, RZ, and YL wrote the first draft of the manuscript. All authors revised and approved the final manuscript.

## Funding

This work was supported by the National Natural Science Foundation of China (31770214) and the Science, Science and Technology Program of Guangzhou (201707010090) and Chang Hungda Science Foundation of Sun Yat-sen University.

## Conflict of Interest

The authors declare that the research was conducted in the absence of any commercial or financial relationships that could be construed as a potential conflict of interest.
