# Supplementary figures and images for: Analyses of Plastome Sequences Improve Phylogenetic Resolution and Provide New Insight Into the Evolutionary History of Asian Sonerileae/Dissochaeteae
Source: Front Plant Sci. 2019 Nov 21;10:1477. doi: 10.3389/fpls.2019.01477 (PMC6881482; doi:10.3389/fpls.2019.01477)

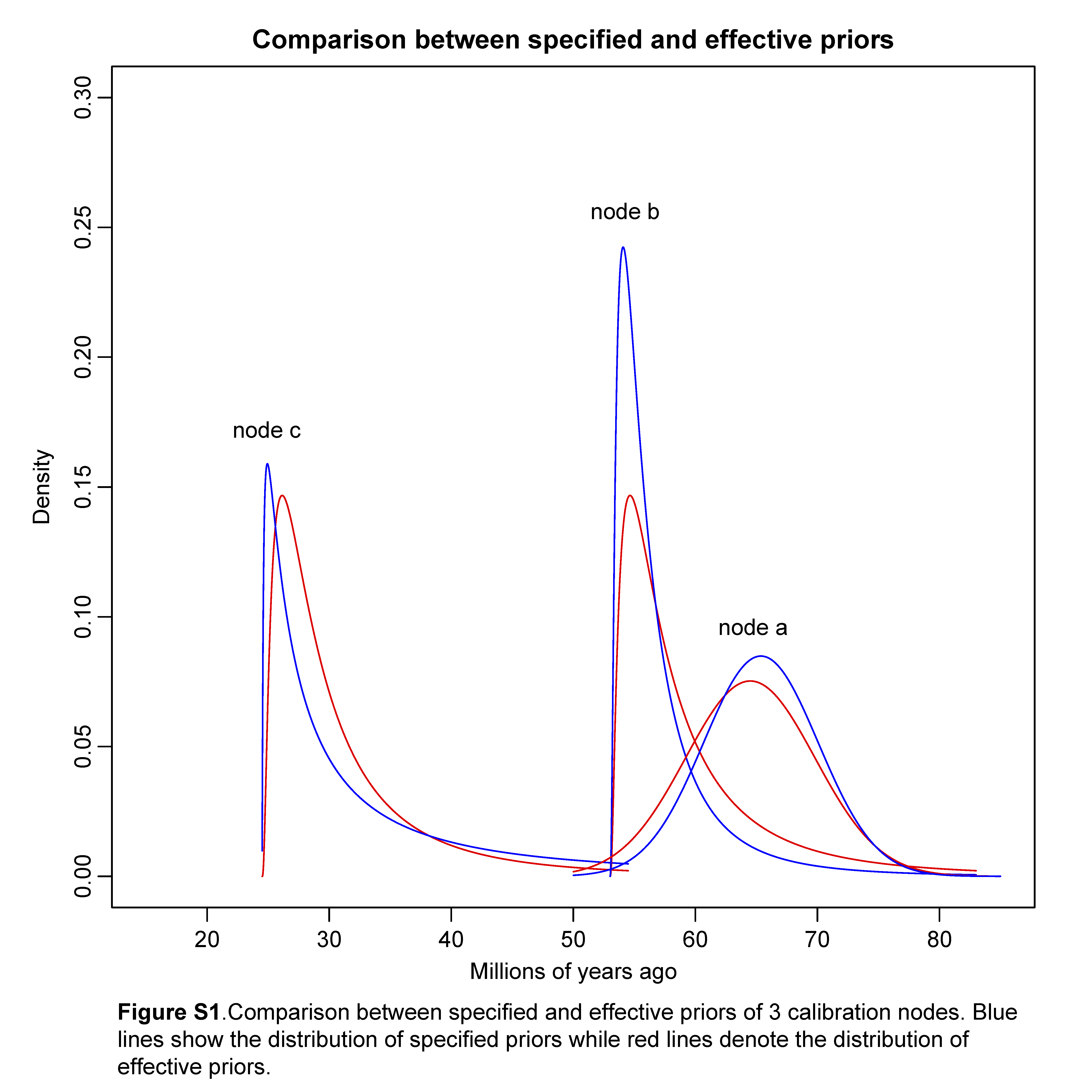

Supplement: Supplementary file 1 [file Image_1.tif]

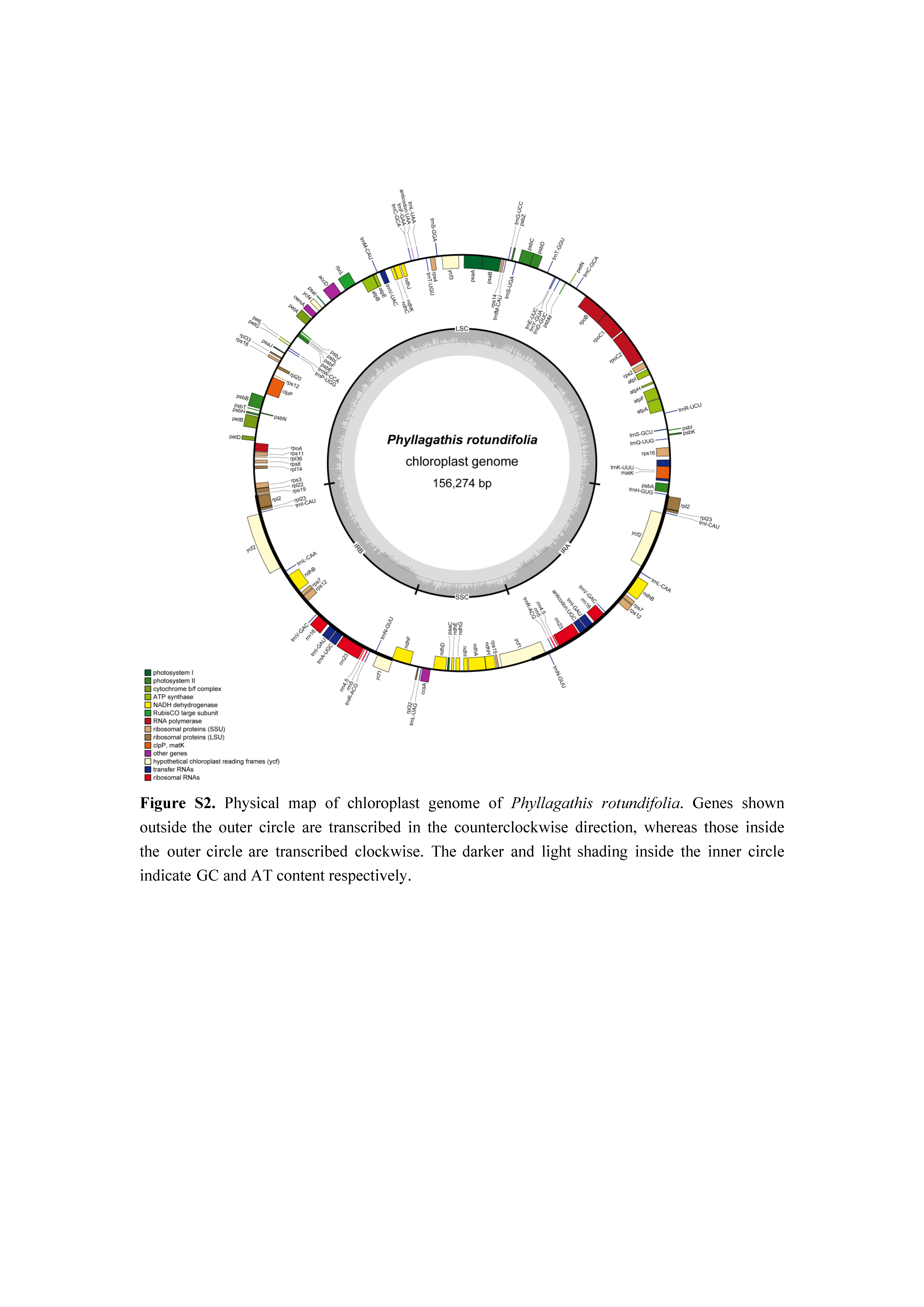

Supplement: Supplementary file 2 [file Image_2.tif]

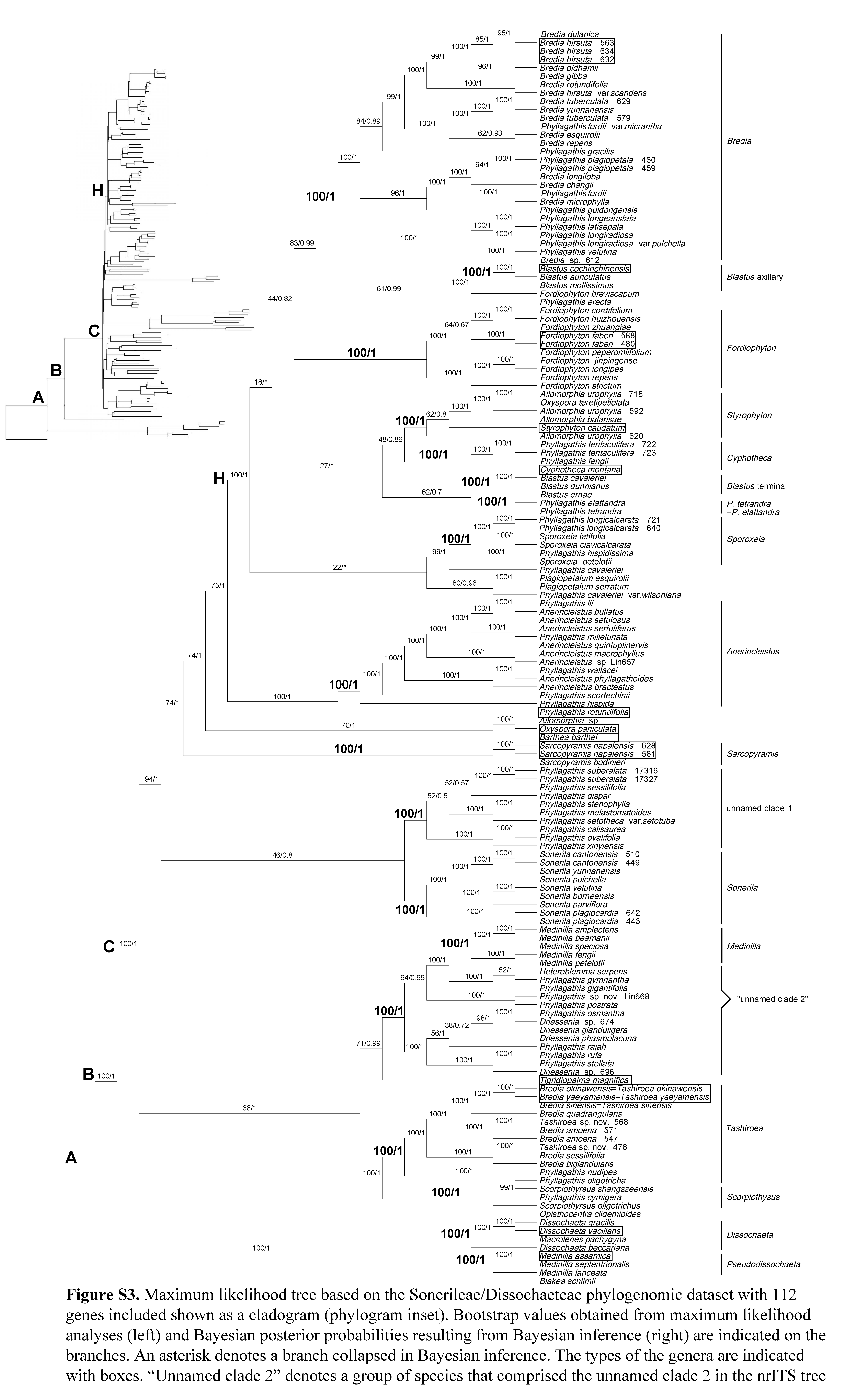

Supplement: Supplementary file 3 [file Image_3.tif]

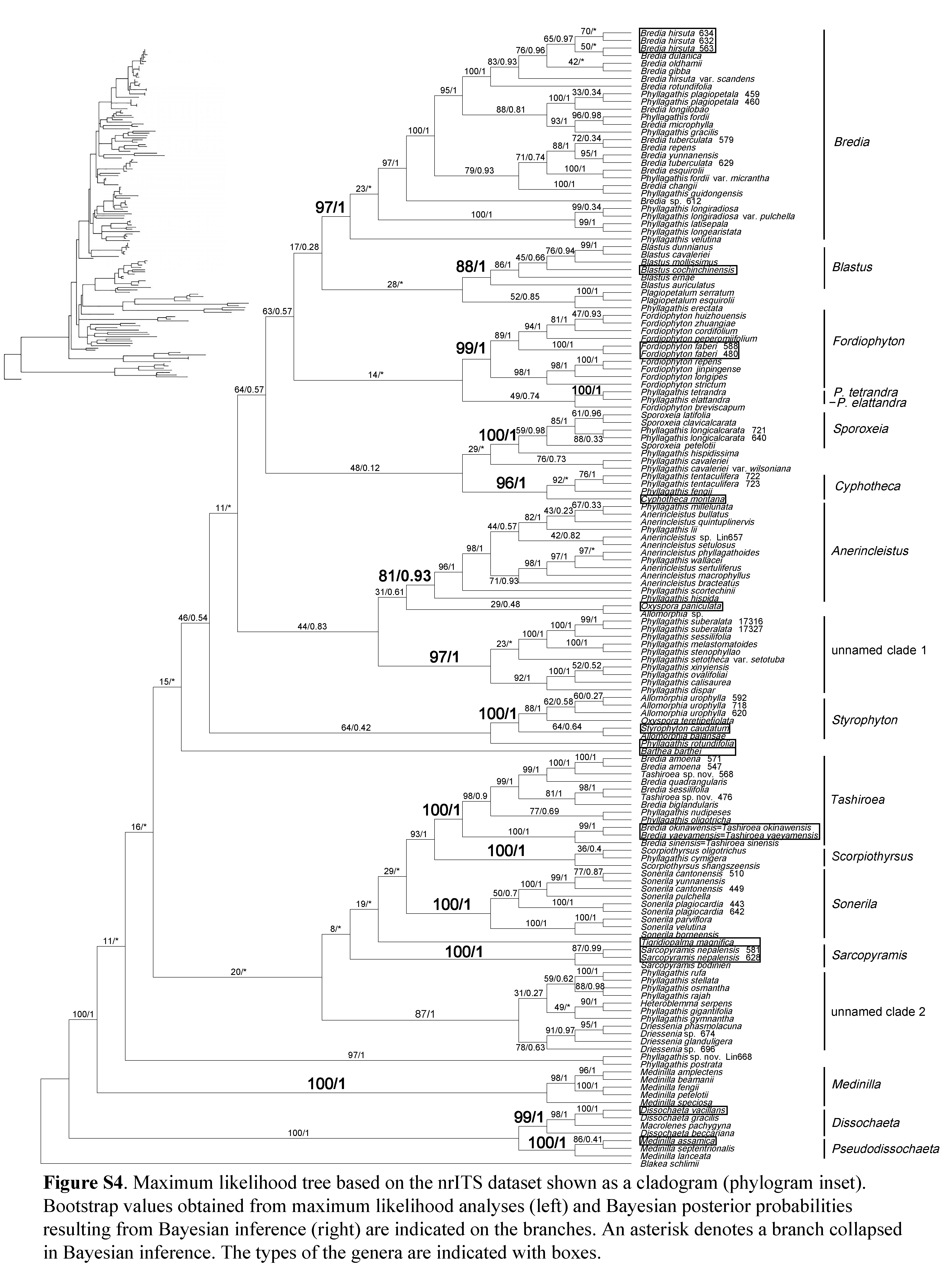

Supplement: Supplementary file 4 [file Image_4.tif]

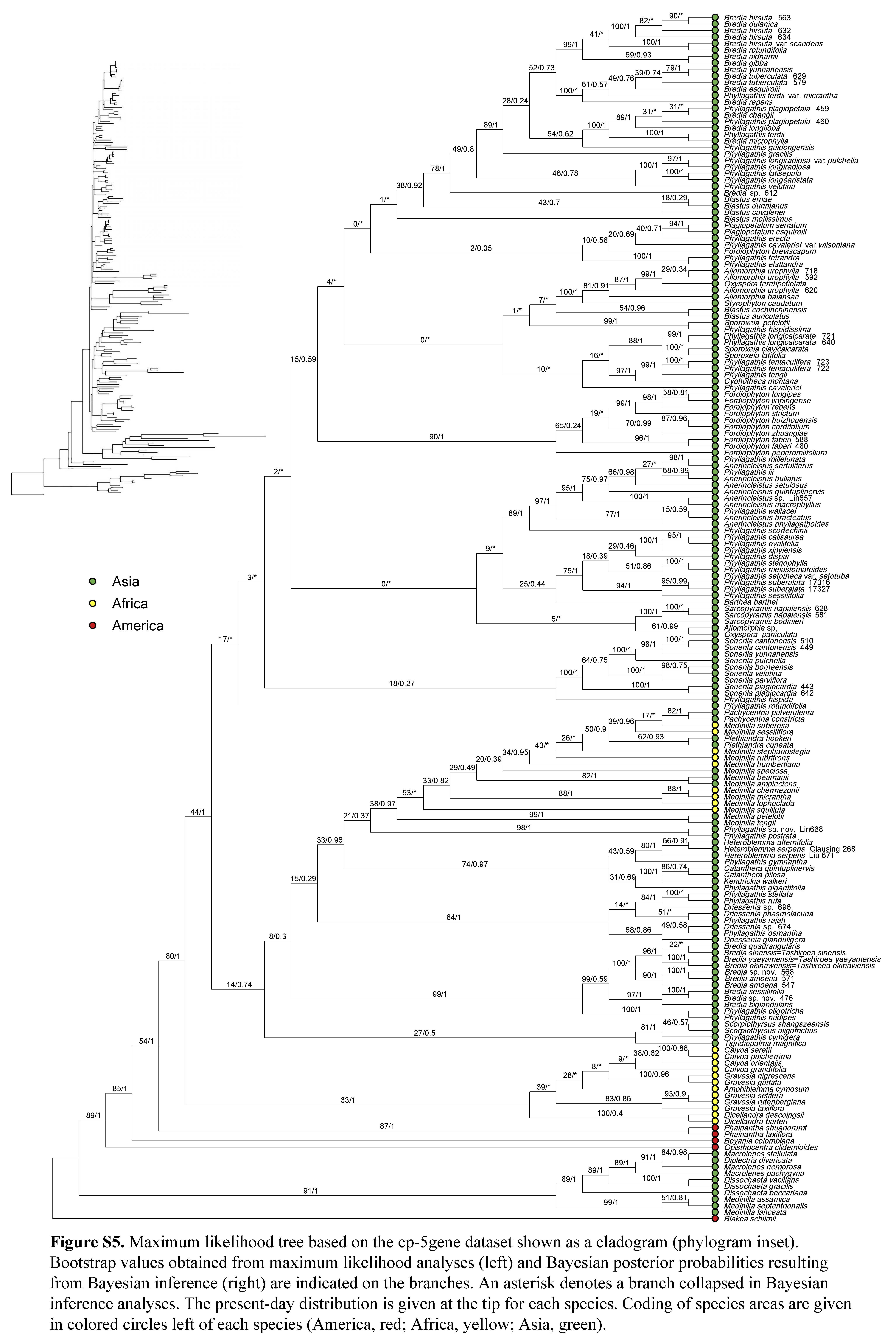

Supplement: Supplementary file 5 [file Image_5.tif]
