# Supplementary material for: Analyses of Plastome Sequences Improve Phylogenetic Resolution and Provide New Insight Into the Evolutionary History of Asian Sonerileae/Dissochaeteae
Source: Front Plant Sci. 2019 Nov 21;10:1477. doi: 10.3389/fpls.2019.01477 (PMC6881482; doi:10.3389/fpls.2019.01477)
Supplement: Supplementary file 6 [file Table_1.docx]

**Table. S1.** Source of materials studied and GenBank accession numbers. Sequences downloaded from GenBank are indicated in bold.

| Taxon | Locality | Voucher | Length (bp) | Accession |
| --- | --- | --- | --- | --- |
| **Myrtaceae** |  |  |  |  |
| *Eucalyptus grandis* W. Mill ex Maiden |  |  | 160, 137 | **HM347959** |
| **Melastomataceae** |  |  |  |  |
| **Tr. Memecyleae** |  |  |  |  |
| *Memecylon ligustrifolium* Champ. ex Benth. | Yingde, Guangdong, China | *Liu 726* (SYS) | 157, 154 | MK994913 |
| **Tr. Kibessieae** |  |  |  |  |
| *Pternandra korthalsiana* Triana. | Sabah, Malaysia | *Zhou et al. 651* (SYS) | 157, 496 | MK994877 |
| *Pternandra tesellata* (Stapf) M.P. Nayar | Sarawak, Malaysia | *Zhou et al. 692* (SYS) | 156, 994 | MK994896 |
| **Tr. Astronieae** |  |  |  |  |
| *Astronia smilacifolia* Triana | Sabah, Malaysia | *Zhou et al. 664* (SYS) | 157, 991 | MK994883 |
| **Tr. Henrietteeae** |  |  |  |  |
| *Henriettea barkeri* (Urb. & Ekman) Alain |  | *G. Ionta 2029* (FLAS) | 156, 527 | **KX826824** |
| **Tr. Merianieae** |  |  |  |  |
| *Graffenrieda moritziana* Triana |  | *F.A. Michelangeli 832* (NY) | 155, 733 | **KX826823** |
| *Salpinga maranonensis* Wurdack |  | *J.L. Clark 13577* (UNA) | 153, 311 | **KX826832** |
| **Tr. Miconieae** |  |  |  |  |
| *Eriocnema fulva* Naudin |  | *F. Almeda 8416* (CAS) | 155, 994 | **KX826822** |
| *Miconia dodecandra* Cogn. |  | *F.A. Michelangeli 758* (NY) | 157, 217 | **KX826826** |
| **Tr. Bertolonieae** |  |  |  |  |
| *Bertolonia acuminata* Gardner |  | *R. Goldenberg 810* (NY, UPCB) | 156, 046 | **KX826820** |
| **Tr. Blakeeae** |  |  |  |  |
| *Blakea* *schlimii* (Naudin) Triana |  | *F.A. Michelangeli 1227* (NY) | 155, 862 | **KX826821** |
| **Tr. Dissochaeteae** |  |  |  |  |
| *Dissochaeta beccariana* Cogn. | Kuching, Malaysia | *Zhou et al. 676* (SYS) | 156, 285 | MK994889 |
| *Dissochaeta gracilis* Blume | Java, Indonesia | *Fan 15704* (SYS) | 156, 227 | MK994855 |
| *Dissochaeta vacillans* Blume | Java, Indonesia | *Fan 15703* (SYS) | 156, 200 | MK994856 |
| *Heteroblemma serpens* (Stapf) Cámara-Leret, Ridd.-Num. & Veldkamp | Sarawak, Malaysia | *Liu 671* (SYS) | 156, 047 | MK994886 |
| *Macrolenes pachygyna* (Korth.) M.P. Nayar | Sarawak, Malaysia | *Zhou et al. 687* (SYS) | 156, 366 | MK994894 |
| *Medinilla amplectens* Regalado | Sabah, Malaysia | *Zhou et al. 663* (SYS) | 155, 529 | MK994882 |
| *Medinilla assamica* (C.B. Clarke) C. Chen | Hekou, Yunnan, China | *Liu 590* (SYS) | 156, 420 | MK994848 |
| *Medinilla beamanii* Regalado | Sabah, Malaysia | *Zhou et al. 658* (SYS) | 155, 271 | MK994880 |
| *Medinilla fengii* (S.Y. Hu) C.Y. Wu & C. Chen | Malipo, Yunnan, China | *Liu 500* (SYS) | 155, 841 | MK994809 |
| *Medinilla lanceata* (M.P. Nayar) C. Chen | Jinping, Yunnan, China | *Liu 593* (SYS) | 156, 790 | MK994850 |
| *Medinilla petelotii* Merr. | Malipo, Yunnan, China | *Liu 589* (SYS) | 155, 673 | MK994847 |
| *Medinilla septentrionalis* (W.W. Sm.) H.L. Li | Malipo, Yunnan, China | *Liu 618* (SYS) | 156, 580 | MK994778 |
| *Medinilla speciosa Blume* | Sabah, Malaysia | *Zhou et al. 669* (SYS) | 155, 084 | MK994885 |
| **Tr. Sonerileae** |  |  |  |  |
| *Allomorphia balansae* Cogn. | Wuzhishan, Hainan, China | *Liu 451* (SYS) | 156, 352 | MK994792 |
| *Allomorphia* sp*.* | Sarawak, Malaysia | *Zhou et al. 681* (SYS) | 155, 328 | MK994891 |
| *Allomorphia urophylla* Diels | Malipo, Yunnan, China | *Liu 592* (SYS) | 156, 299 | MK994849 |
| *Allomorphia urophylla* Diels | Malipo, Yunnan, China | *Liu 620* (SYS) | 156, 162 | MK994861 |
| *Allomorphia urophylla* Diels | Jinping, Yunnan, China | *Liu 718* (SYS) | 156, 317 | MK994903 |
| *Anerincleistus bracteatus* C. Hansen | Sarawak, Malaysia | *Zhou et al. 698* (SYS) | 156, 862 | MK994899 |
| *Anerincleistus bullatus* J.F. Maxwell | Sarawak, Malaysia | *Zhou et al. 695* (SYS) | 156, 823 | MK994897 |
| *Anerincleistus macrophyllus* Bakh.f. | Sarawak, Malaysia | *Zhou et al. 673* (SYS) | 156, 903 | MK994887 |
| *Anerincleistus phyllagathoides* (Stapf) J.F. Maxwell | Sarawak, Malaysia | *C.W. Lin 635* (TAIF) | 156, 884 | MK994917 |
| *Anerincleistus quintuplinervis* (Cogn.) J.F. Maxwell | Sabah, Malaysia | *Zhou et al. 654* (SYS) | 157, 119 | MK994878 |
| *Anerincleistus sertuliferus* (Cogn.) J.F. Maxwell | Sarawak, Malaysia | *Zhou et al. 675* (SYS) | 157, 069 | MK994888 |
| *Anerincleistus setulosus* O.Schwartz | Sabah, Malaysia | *Zhou et al. 660* (SYS) | 156, 523 | MK994881 |
| *Anerincleistus* sp*.* | Sarawak, Malaysia | *C.W. Lin 657* (TAIF) | 156, 934 | MK994922 |
| *Barthea barthei* (Hance ex Benth.) Krasser | Shenzhen, China | *Y.C. Cai s.n.* (SYS) | 155, 948 | MK994907 |
| *Blastus auriculatus* Y.C. Huang | Hekou, Nanxi, China | *Liu 542* (SYS) | 155, 981 | **MK335944** |
| *Blastus cavaleriei* H. Lév. & Vaniot | Dongan, Hunan, China | *Liu 461* (SYS) | 155, 999 | MK994908 |
| *Blastus cochinchinensis* Lour. | Fengkai, Guandong, China | *Liu 446* (SYS) | 155, 969 | MK994909 |
| *Blastus dunnianus* H. Lév. | Pingnan, Guangxi, China | *Liu 477* (SYS) | 156, 004 | MK994804 |
| *Blastus ernae* Hand.-Mazz. | Rucheng, Hunan, China | *Liu 469* (SYS) | 155, 994 | MK994800 |
| *Blastus mollissimus* H.L. Li | Guiping, Guangxi, China | *Liu 622* (SYS) | 155, 410 | MK994863 |
| *Bredia amoena* Diels | Zixi, Jiangxi, China | *Liu 547* (SYS) | 156, 101 | MK994826 |
| *Bredia amoena* Diels | Pingnan, Fujian, China | *Liu 571* (SYS) | 155, 915 | MK994838 |
| *Bredia biglandularis* C. Chen | Fangcheng, Guangxi, China | *Liu 553* (SYS) | 156, 298 | MK994829 |
| *Bredia changii* W.Y. Zhao, X.H. Zhan & W.B. Liao | Chongyi, Jiangxi, China | *Liu 548* (SYS) | 156, 066 | MK994827 |
| *Bredia dulanica* C.L. Yeh, S.W. Chung & T.C. Hsu | Dulanshan, Taidong, Taiwan | *Liu 565* (SYS) | 155, 222 | MK994834 |
| *Bredia esquirolii* (H. Lév.) Lauener | Chishui, Guizhou, China | *Liu 587* (SYS) | 155, 957 | MK994845 |
| *Bredia gibba* Ohwi | Pingdong, Taiwan | *Liu 566* (SYS) | 155, 896 | MK994835 |
| *Bredia hirsuta* Blume | Komi, Iriomote, Japan | *Liu 632* (SYS) | 155, 707 | MK994871 |
| *Bredia hirsuta* Blume | Okinawa, Japan | *Liu 634* (SYS) | 155, 707 | MK994872 |
| *Bredia hirsuta* Blume | Taidong, Taiwan | *Liu 563* (SYS) | 155, 713 | MK994833 |
| *Bredia hirsuta* var. *scandens* Ito & Matsum. | Nantou, Taiwan | *Liu 539* (SYS) | 155, 733 | MK994823 |
| *Bredia longiloba* (Hand.-Mazz.) Diels | Zixi, Jiangxi, China | *Liu 544* (SYS) | 156, 166 | MK994825 |
| *Bredia microphylla* H.L. Li | Longsheng, Guangxi, China | *Liu 551* (SYS) | 156, 193 | MK994828 |
| *Bredia okinawensis* (Matsumura) H.L. Li (=*Tashiroea okinawensis* Matsum.) | Okinawa, Japan | *Liu 636* (SYS) | 156, 023 | MK994873 |
| *Bredia oldhamii* Hook. f. | Pingdong, Taiwan | *Liu 533* (SYS) | 155, 885 | MK994821 |
| *Bredia quadrangularis* Cogn. | Guidong, Hunan, China | *Liu 473* (SYS) | 156, 005 | MK994802 |
| *Bredia repens* R.C. Zhou, Q.J. Zhou & Y. Liu | Sangzhi, Hunan, China | *Liu 558* (SYS) | 156, 063 | MK994832 |
| *Bredia rotundifolia* Y.C. Liu & C.H. Ou | Zhanghua, Taiwan | *Liu 538* (SYS) | 156, 090 | MK994822 |
| *Bredia sessilifolia* H.L. Li | Huaiji, Guangdong, China | *Liu 540* (SYS) | 156, 104 | MK994824 |
| *Bredia sinensis* (Diels) H.L. Li (=*Tashiroea sinensis* Diels) | Pingnan, Fujian, China | *Liu 569* (SYS) | 155, 994 | MK994837 |
| *Bredia* sp*.* | Jinping, Yunnan, China | *Liu 612* (SYS) | 156, 116 | MK994859 |
| *Bredia tuberculata* (Guillaumin) Diels | Emei, Sichuan, China | *Liu 579* (SYS) | 155, 953 | MK994841 |
| *Bredia tuberculata* (Guillaumin) Diels | Shuifu, Yunnan, China | *Liu 629* (SYS) | 155, 983 | MK994869 |
| *Bredia yaeyamensis* (Matsum.) H.L. Li (=*Tashiroea yaeyamensis* Matsum.) | Iriomote, Japan | *Liu 631* (SYS) | 156, 147 | MK994870 |
| *Bredia yunnanensis* (H.Lév.) Diels | Shuigu, Yunnan, China | *Liu 627* (SYS) | 155, 973 | MK994867 |
| *Cyphotheca montana* Diels | Jinping, Yunnan, China | *Liu 596* (SYS) | 156, 422 | MK994852 |
| *Driessenia glanduligera* Stapf | Sabah, Malaysia | *Zhou et al. 657* (SYS) | 156, 509 | MK994879 |
| *Driessenia phasmolacuna* C.W. Lin | Sarawak, Malaysia | *C.W. Lin 659* (SYS) | 156, 620 | MK994923 |
| *Driessenia* sp. | Sarawak, Malaysia | *Zhou et al. 674* (SYS) | 156, 674 | MK994780 |
| *Driessenia* sp*.* | Sarawak, Malaysia | *Zhou et al. 696* (SYS) | 156, 142 | MK994898 |
| *Fordiophyton breviscapum* (C. Chen) Y.F. Deng & T.L. Wu | Ruyuan, Guangdong, China | *Liu 441* (SYS) | 155, 874 | MK994788 |
| *Fordiophyton cordifolium* C.Y. Wu ex C. Chen | Gaozhou, Guangdong, China | *Liu 430* (SYS) | 155, 632 | MK994784 |
| *Fordiophyton faberi* Stapf | Pingnan, Guangxi, China | *Liu 480* (SYS) | 155, 543 | MK994805 |
| *Fordiophyton faberi* Stapf | Emei, Sichuan, China | *Liu 588* (SYS) | 155, 728 | MK994846 |
| *Fordiophyton huizhouense* S.J. Zeng & X.Y. Zhuang | Huidong, Guangdong, China | *Liu 433* (SYS) | 155, 639 | MK994786 |
| *Fordiophyton jinpingense* J.H. Dai & Z.Y. Yu | Jinping, Yunnan, China | *Liu 641* (SYS) | 154, 430 | MK994875 |
| *Fordiophyton longipes* Y.C. Huang | Pingbian, Yunnan, China | *Liu 610* (SYS) | 154, 928 | MK994858 |
| *Fordiophyton peperomiifolium* (Oliv.) C. Hansen | Qingyuan, Guangdong, China | *Liu 432* (SYS) | 154, 453 | MK994785 |
| *Fordiophyton repens* Y.C. Huang ex C. Chen | Pingbian, Yunnan, China | *Liu 513* (SYS) | 154, 970 | MK994815 |
| *Fordiophyton strictum* Diels | Pingbian, Yunnan, China | *Liu 514* (SYS) | 155, 110 | MK994816 |
| *Fordiophyton zhuangiae* S.J. Zeng & G.D. Tang | Yangchuan, Guangdong, China | *Liu 574* (SYS) | 155, 423 | MK994839 |
| *Ochthocharis bornensis* Blume | Sarawak, Malaysia | *Zhou et al. 689* (SYS) | 156, 672 | MK994895 |
| *Opisthocentra clidemioides* Hook.f. |  | *M.K. Caddah 578* (NY, UPCB) | 156, 352 | **KX826828** |
| *Oxyspora paniculata* DC. | Malipo, Yunnan, China | *Liu 523* (SYS) | 156, 331 | MK994819 |
| *Oxyspora teretipetiolata* (C.Y. Wu & C. Chen) W.H. Chen & Y.M. Shui | Jinping, Yunnan, China | *Liu 598* (SYS) | 156, 303 | MK994853 |
| *Phyllagathis calisaurea* C. Chen | Napo, Guangxi, China | *Liu 625* (SYS) | 155, 458 | MK994865 |
| *Phyllagathis cavaleriei* (H. Lév. & Vaniot) Guillaumin | Dongkou, Hunan, China | *Liu 456* (SYS) | 156, 406 | MK994795 |
| *Phyllagathis cavaleriei* var. *wilsoniana* Guillaumin | Hongya, Sichuan, China | *Liu 599* (SYS) | 155, 868 | MK994854 |
| *Phyllagathis cymigera* C. Chen | Malipo, Yunnan, China | *Liu 624* (SYS) | 156, 317 | MK994864 |
| *Phyllagathis dispar* (Cogn.) C. Hansen | Sabah, Malaysia | *Zhou et al. M20* (SYS) | 155, 978 | MK994910 |
| *Phyllagathis elattandra* Diels | Guiping, Guangxi, China | *Liu 554* (SYS) | 155, 370 | MK994830 |
| *Phyllagathis erecta* (S.Y. Hu) C.Y. Wu ex C. Chen | Malipo, Yunnan, China | *Liu 507* (SYS) | 156, 360 | MK994811 |
| *Phyllagathis fengii* C. Hansen | Xichou, Yunnan, China | *Liu 520* (SYS) | 156, 336 | MK994818 |
| *Phyllagathis fordii* (Hance) C. Chen | Fengkai, Guangdong, China | *Liu 444* (SYS) | 156, 154 | MK994790 |
| *Phyllagathis fordii* (Hance) C. Chen var. *micrantha* C. Chen | Leshan, Sichuan, China | *Liu 580* (SYS) | 155, 952 | MK994842 |
| *Phyllagathis gigantifolia* M.P. Nayar | Sabah, Malaysia | *Zhou et al. 659* (SYS) | 156, 350 | MK994779 |
| *Phyllagathis gracilis* (Hand.-Mazz.) C. Chen | Wugang, Hunan, China | *Liu 457* (SYS) | 155, 894 | MK994796 |
| *Phyllagathis guidongensis* K.M. Liu & J. Tian | Guidong, Hunan, China | *Liu 472* (SYS) | 155, 853 | MK994801 |
| *Phyllagathis gymnantha* Korth. | Sarawak, Malaysia | *C.W. Lin 625* (TAIF) | 156, 607 | MK994918 |
| *Phyllagathis hispida* King | Kuala Lumpur, Malaysia | *Zhou et al. M49* (SYS) | 156, 365 | MK994783 |
| *Phyllagathis hispidissima* (C. Chen) C. Chen | Pingbian, Yunnan, China | *Liu 604* (SYS) | 156, 166 | MK994857 |
| *Phyllagathis latisepala* C. Chen | Sangzhi, Hunan, China | *Liu 557* (SYS) | 155, 744 | MK994831 |
| *Phyllagathis lii* C.W. Lin, Chien F. Chen & T.Y.A. Yang | Sarawak, Malaysia | *C.W. Lin 667* (TAIF) | 156, 958 | MK994924 |
| *Phyllagathis longearistata* C. Chen | Huanjiang, Guangxi, China | *Liu 498* (SYS) | 156, 026 | MK994808 |
| *Phyllagathis longiradiosa* (C. Chen) C. Chen | Longzhou, Guangxi, China | *Liu 486* (SYS) | 155, 849 | MK994807 |
| *Phyllagathis longiradiosa* var. *pulchella* C. Chen | Longzhou, Guangxi, China | *Liu 485* (SYS) | 155, 871 | MK994806 |
| *Phyllagathis longicalcarata* C. Hansen | Jinping, Yunnan, China | *Liu 640* (SYS) | 156, 199 | MK994874 |
| *Phyllagathis longicalcarata* C. Hansen | Jinping, Yunnan, China | *Liu 721* (SYS) | 156, 232 | MK994905 |
| *Phyllagathis melastomatoides* (Merr. & Chun) W.C. Ko | Lingshui, Hainan, China | *Liu 447* (SYS) | 155, 822 | MK994914 |
| *Phyllagathis millelunata* C.W. Lin, Chien F. Chen & T.Y.A. Yang | Sarawak, Malaysia | *C.W. Lin 582* (TAIF) | 157, 069 | MK994916 |
| *Phyllagathis nudipes* C. Chen | Ruyuan, Guangdong, China | *Liu 435* (SYS) | 154, 453 | MK994787 |
| *Phyllagathis oligotricha* Merr. | Rucheng, Hunan, China | *Liu 468* (SYS) | 156, 278 | MK994799 |
| *Phyllagathis osmantha* (M.P. Nayar) Cellin. | Sarawak, Malaysia | *C.W. Lin 567* (TAIF) | 156, 987 | MK994915 |
| *Phyllagathis ovalifolia* H.L. Li | Pingbian, Yunnan, China | *Liu 512* (SYS) | 155, 632 | MK994814 |
| *Phyllagathis plagiopetala* C. Chen | Xinning, Hunan, China | *Liu 459* (SYS) | 156, 270 | MK994797 |
| *Phyllagathis plagiopetala* C. Chen | Dongan, Hunan, China | *Liu 460* (SYS) | 156, 237 | MK994798 |
| *Phyllagathis postrata* C. Hansen | Gia Lai Province, Vietnam | *C.W. Lin 640* (TAIF) | 155, 851 | MK994919 |
| *Phyllagathis rajah* C.W. Lin, Chien F. Chen & T.Y.A. Yang | Sarawak, Malaysia | *C.W. Lin 644* (TAIF) | 156, 105 | MK994921 |
| *Phyllagathis rotundifolia* (Jack) Blume | Kuala Lumpur, Malasysia | *Zhou et al. M50* (SYS) | 156, 274 | MK994912 |
| *Phyllagathis rufa* (Stapf) Cellin. | Sarawak, Malaysia | *Zhou et al. 679* (SYS) | 157, 556 | MK994890 |
| *Phyllagathis scortechinii* King | Kuala Lumpur, Malasysia | *Zhou et al. M48* (SYS) | 156, 108 | MK994911 |
| *Phyllagathis sessilifolia* C.Hansen | Vietnam | *Q. Fan 17311* (SYS) | 156, 038 | MK994926 |
| *Phyllagathis setotheca* var. *setotuba* C. Chen | Yangchun, Guangzhou, China | *Liu 576* (SYS) | 156, 158 | MK994840 |
| *Phyllagathis* sp. nov*.* | Vietnam | *C.W. Lin 668* (TAIF) | 155, 318 | MK994925 |
| *Phyllagathis stellata* C.W. Lin & C.H. Lee | Sarawak, Malaysia | *C.W. Lin 643* (TAIF) | 158, 960 | MK994920 |
| *Phyllagathis stenophylla* (Merr. & Chun) H.L. Li | Ledong, Hainan, China | *Liu 453* (SYS) | 155, 832 | MK994793 |
| *Phyllagathis suberalata* C.Hansen | Vietnam | *Q. Fan 17316* (SYS) | 156, 068 | MK994927 |
| *Phyllagathis suberalata* C.Hansen | Vietnam | *Q. Fan 17327* (SYS) | 156, 075 | MK994928 |
| *Phyllagathis tentaculifera* C.Hansen | Jinping, Yunnan, China | *Liu 722* (SYS) | 156, 440 | MK994782 |
| *Phyllagathis tentaculifera* C.Hansen | Jinping, Yunnan, China | *Liu 723* (SYS) | 156, 445 | MK994906 |
| *Phyllagathis tetrandra* Diels | Xichou, Yunnan, China | *Liu 519* (SYS) | 154, 818 | MK994817 |
| *Phyllagathis velutina* (Diels) C. Chen | Malipo, Yunnan, China | *Liu 509* (SYS) | 156, 001 | MK994812 |
| *Phyllagathis wallacei* C.W. Lin, Chien F. Chen & T.Y.A. Yang | Sarawak, Malaysia | *Zhou et al. 686* (SYS) | 156, 719 | MK994781 |
| *Phyllagathis xinyiensis* Z.J. Feng | Xinyi, Guangdong, China | *Liu 582* (SYS) | 155, 462 | MK994844 |
| *Plagiopetalum esquirolii* (H. Lév.) Rehder | Malipo, Yunnan, China | *Liu 594* (SYS) | 155, 637 | MK994851 |
| *Plagiopetalum serratum* (Diels) Diels | Jinping, Yunnan, China | *Liu 717* (SYS) | 156, 181 | MK994902 |
| *Sarcopyramis bodinieri* H. Lév. | Malipo, Yunnan, China | *Liu 502* (SYS) | 153, 312 | MK994810 |
| *Sarcopyramis nepalensis* Wall. | Zhangjiajie, Hunan, China | *Liu 581* (SYS) | 153, 291 | MK994843 |
| *Sarcopyramis nepalensis* Wall. | Shuifu, Yunnan, China | *Liu 628* (SYS) | 153, 304 | MK994868 |
| *Scorpiothyrsus oligotrichus* H.L. Li | Ledong, Hainan, China | *Liu 454* (SYS) | 156, 105 | MK994794 |
| *Scorpiothyrsus shangszeensis* C. Chen | Napo, Guangxi, China | *Liu 626* (SYS) | 156, 371 | MK994866 |
| *Sonerila borneensis* Cogn. | Sarawak, Malaysia | *Zhou et al. 684* (SYS) | 154, 804 | MK994893 |
| *Sonerila cantonensis* Stapf | Malipo, Yunnan, China | *Liu 510* (SYS) | 155, 233 | MK994813 |
| *Sonerila cantonensis* Stapf | Lingshui, Hainan, China | *Liu 449* (SYS) | 155, 265 | MK994791 |
| *Sonerila parviflora* Cogn. | Sarawak, Malaysia | *Zhou et al. 700* (SYS) | 154, 752 | MK994900 |
| *Sonerila plagiocardia* Diels | Yingde, Guangdong, China | *Liu 443* (SYS) | 153, 922 | MK994789 |
| *Sonerila plagiocardia* Diels | Jinping, Yunnan, China | *Liu 642* (SYS) | 154, 428 | MK994876 |
| *Sonerila pulchella* Stapf | Sabah, Malaysia | *Zhou et al. 668* (SYS) | 154, 862 | MK994884 |
| *Sonerila velutina* Cogn. | Sarawak, Malaysia | *Zhou et al. 683* (SYS) | 154, 682 | MK994892 |
| *Sonerila yunnanensis* Jeffrey ex W.W. Sm. | Malipo, Yunnan, China | *Liu 621* (SYS) | 154, 964 | MK994862 |
| *Sporoxeia clavicalcarata* C. Chen | Jinping, Yunnan, China | *Liu 716* (SYS) | 156, 144 | MK994901 |
| *Sporoxeia latifolia* (H.L.Li) C.Y. Wu & Y.C. Huang | Malipo, Yunnan, China | *Liu 524* (SYS) | 156, 188 | MK994820 |
| *Sporoxeia petelotii* (Merr.) C. Hansen | Jinping, Yunnan, China | *Liu 719* (SYS) | 156, 529 | MK994904 |
| *Styrophyton caudatum* (Diels) S.Y. Hu | Malipo, Yunnan, China | *Liu 615* (SYS) | 156, 386 | MK994860 |
| *Tashiroea* sp. nov*.* | Pingnan, Guangxi, China | *Liu 476* (SYS) | 156, 263 | MK994803 |
| *Tashiroea* sp. nov*.* | Pingnan, Fujian, China | *Liu 568* (SYS) | 156, 004 | MK994836 |
| *Tigridiopalma magnifica* C. Chen | Gaozhou, Guangdong, China | *Liu 429* (SYS) | 155, 663 | **MF663760** |
| **Tr. Microlicieae** |  |  |  |  |
| *Rhynchanthera bracteata* Triana |  | *F. Zenteno 8801* (NY) | 155, 108 | **KX826831** |
| **Tr. Rhexieae** |  |  |  |  |
| *Rhexia virginica* L. |  | *F.A. Michelangeli 1448* (NY) | 154, 635 | **KX826830** |
| **Tr. Melastomateae** |  |  |  |  |
| *Melastoma candidum* D. Don | Wenchang, Hainan, China |  | 156, 682 | **KY745894** |
| *Nepsera aquatica* Naudin |  | *F.A. Michelangeli 1998* (NY) | 155, 110 | **KX826827** |
| *Pterogastra divaricata* (Bonpl.) Naudin |  | *F.A. Michelangeli 540* (NY) | 154, 948 | **KX826829** |
| *Tibouchina longifolia* Baill. |  | *L. Majure 4277* (FLAS) | 156, 789 | **KX826833** |
| **Merianthera Group** |  |  |  |  |
| *Merianthera pulchra* Kuhlm. |  | *R. Goldenberg 1153* (NY, UPCB) | 156, 168 | **KX826825** |
| **Tr. Cyphostyleae** |  |  |  |  |
| *Allomaieta villosa* (Gleason) Lozano |  | *H. David 2188* (HUA, NY) | 156, 452 | **KX826819** |
| **Tr. Trioieneae** |  |  |  |  |
| *Triolena amazonica* (Pilg.) Wurdack |  | *F.A. Michelangeli 1366* (NY) | 156, 652 | **KX826834** |
