# Supplementary material for: Analyses of Plastome Sequences Improve Phylogenetic Resolution and Provide New Insight Into the Evolutionary History of Asian Sonerileae/Dissochaeteae
Source: Front Plant Sci. 2019 Nov 21;10:1477. doi: 10.3389/fpls.2019.01477 (PMC6881482; doi:10.3389/fpls.2019.01477)
Supplement: Supplementary file 7 [file Table_2.docx]

**Table S2.** Source of materials studied and GenBank accession numbers for nrITS and 5 chloroplast intergenic spacer/genes. Sequences downloaded from GenBank are indicated in bold. Hash signs denote the sequences directly extracted from complete chloroplast genome.

| **Species** | **Voucher** | **accD** | **psbK-psbL** | **ndhF** | **rbcL** | **rpl16** | **ITS** |
| --- | --- | --- | --- | --- | --- | --- | --- |
| *Allomorphia balansae* Cogn. | *Liu 451* (SYS) | # | # | # | # | # | **MG644470** |
| *Allomorphia* sp*.* | *Zhou et al. 681* (SYS) | # | # | # | # | # | MN031168 |
| *Allomorphia urophylla* Diels | *Liu 592* (SYS) | # | # | # | # | # | MN031230 |
| *Allomorphia urophylla* Diels | *Liu 620* (SYS) | # | # | # | # | # | MN031227 |
| *Allomorphia urophylla* Diels | *Liu 718* (SYS) | # | # | # | # | # | MN031167 |
| *Amphiblemma cymosum* Naudin | *cult. BG Mainz* | - | - | **AF215588** | **AF215543** | **AF215624** | - |
| *Anerincleistus bracteatus* C .Hansen | *Zhou et al. 698* (SYS) | # | # | # | # | # | MN031178 |
| *Anerincleistus bullatus* J.F. Maxwell | *Zhou et al. 695* (SYS) | # | # | # | # | # | MN031179 |
| *Anerincleistus macrophyllus* Bakh.f. | *Zhou et al. 673* (SYS) | # | # | # | # | # | MN031184 |
| *Anerincleistus phyllagathoides* (Stapf) J.F. Maxwell | *C.W. Lin 635* (TAIF) | # | # | # | # | # | MN031205 |
| *Anerincleistus quintuplinervis* (Cogn.) J.F. Maxwell | *Zhou et al. 654* (SYS) | # | # | # | # | # | MN031183 |
| *Anerincleistus sertuliferus* (Cogn.) J.F. Maxwell | *Zhou et al. 675* (SYS) | # | # | # | # | # | MN031181 |
| *Anerincleistus setulosus* O. Schwartz | *Zhou et al. 660* (SYS) | # | # | # | # | # | MN031185 |
| *Anerincleistus* sp*.* | *C.W. Lin 657* (TAIF) | # | # | # | # | # | MN031197 |
| *Barthea barthei* (Hance ex Benth.) Krasser | *Y.C. Cai s.n.* (SYS) | # | # | # | # | # | MN031159 |
| *Blakea schlimii* (Naudin) Triana | *F.A. Michelangeli 1227 (NY)* | # | # | # | # | # | **AY460441** |
| *Blastus auriculatus* Y.C.Huang | *Liu 542* (SYS) | # | # | # | # | # | **MG644463** |
| *Blastus cavaleriei* H. Lév. & Vaniot | *Liu 461* (SYS) | # | # | # | # | # | **MG644464** |
| *Blastus cochinchinensis* Lour. | *Liu 446* (SYS) | # | # | # | # | # | **MG644465** |
| *Blastus dunnianus* H. Lév. | *Liu 477* (SYS) | # | # | # | # | # | MN031229 |
| *Blastus ernae* Hand.-Mazz. | *Liu 469* (SYS) | # | # | # | # | # | **MG644468** |
| *Blastus mollissimus* H.L. Li | *Liu 622* (SYS) | # | # | # | # | # | MN031195 |
| *Boyania colombiana* Humberto Mend. | *Daly 14243 (NY)* | - | - | - | **JQ899086** | **JQ899062** | - |
| *Bredia amoena* Diels | *Liu 547* (SYS) | # | # | # | # | # | MN031215 |
| *Bredia amoena* Diels | *Liu 571* (SYS) | # | # | # | # | # | **MG644390** |
| *Bredia biglandularis* C. Chen | *Liu 553* (SYS) | # | # | # | # | # | **MG644392** |
| *Bredia changii* W.Y. Zhao, X.H. Zhan & W.B. Liao | *Liu 548* (SYS) | # | # | # | # | # | **MF952714** |
| *Bredia dulanica* C.L. Yeh, S.W. Chung & T.C. Hsu | *Liu 565* (SYS) | # | # | # | # | # | MN031225 |
| *Bredia esquirolii* (H. Lév.) Lauener | *Liu 587* (SYS) | # | # | # | # | # | **MG644399** |
| *Bredia gibba* Ohwi | *Liu 566* (SYS) | # | # | # | # | # | **MG644415** |
| *Bredia hirsuta* Blume | *Liu 632* (SYS) | # | # | # | # | # | MN031196 |
| *Bredia hirsuta* Blume | *Liu 634* (SYS) | # | # | # | # | # | MN031213 |
| *Bredia hirsuta* Blume | *Liu 563* (SYS) | # | # | # | # | # | **MG644417** |
| *Bredia hirsuta* var. *scandens* Ito & Matsum. | *Liu 539* (SYS) | # | # | # | # | # | **MG644420** |
| *Bredia longiloba* (Hand.-Mazz.) Diels | *Liu 544* (SYS) | # | # | # | # | # | **MF952715** |
| *Bredia microphylla* H.L. Li | *Liu 551* (SYS) | # | # | # | # | # | **MF952716** |
| *Bredia okinawensis* (Matsumura) H.L. Li (=*Tashiroea okinawensis* Matsum.) | *Liu 636* (SYS) | # | # | # | # | # | MN031211 |
| *Bredia oldhamii* Hook. f. | *Liu 533* (SYS) | # | # | # | # | # | MN031237 |
| *Bredia quadrangularis* Cogn. | *Liu 473* (SYS) | # | # | # | # | # | **MG644391** |
| *Bredia repens* R.C. Zhou, Q.J. Zhou & Y. Liu | *Liu 558* (SYS) | # | # | # | # | # | **MF952713** |
| *Bredia rotundifolia* Y.C. Liu & C.H. Ou | *Liu 538* (SYS) | # | # | # | # | # | **MG644419** |
| *Bredia sessilifolia* H.L. Li | *Liu 540* (SYS) | # | # | # | # | # | **MG644393** |
| *Bredia sinensis* (Diels) H.L. Li (=*Tashiroea sinensis* Diels) | *Liu 569* (SYS) | # | # | # | # | # | **MG644398** |
| *Bredia* sp*.* | *Liu 612* (SYS) | # | # | # | # | # | MN031165 |
| *Bredia tuberculata* (Guillaumin) Diels | *Liu 579* (SYS) | # | # | # | # | # | **MG644412** |
| *Bredia tuberculata* (Guillaumin) Diels | *Liu 629* (SYS) | # | # | # | # | # | MN031226 |
| *Bredia yaeyamensis* (Matsum.) H.L. Li (=*Tashiroea yaeyamensis* Matsum.) | *Liu 631* (SYS) | # | # | # | # | # | MN031210 |
| *Bredia yunnanensis* (H. Lév.) Diels | *Liu 627* (SYS) | # | # | # | # | # | MN031189 |
| *Calvoa grandifolia* Cogn. | *Figueiredo 202 ( LISC)* | - | - | - | **AY667151** | **AY660632** | - |
| *Calvoa orientalis* Taub. | *C. Orava 1 (MJG)* | - | - | **AF215589** | **AF215544** | - | - |
| *Calvoa pulcherrima* H. Perr. | *-* | **MG702538** | - | **MG702549** | - | - | - |
| *Calvoa seretii* De Wild. | *-* | **MG702539** | **MG518555** | **MG702550** | - | - | - |
| *Catanthera pilosa* M.P. Nayar | *Clausing 258 (MJG)* | - | - | **AF289367** | - | - | - |
| *Catanthera quintuplinervis* (Cogn.) Nayar | *Clausing 196 (MJG)* | - | - | **AF289368** | - | - | - |
| *Cyphotheca montana* Diels | *Liu 596* (SYS) | # | # | # | # | # | **MG644447** |
| *Dicellandra barteri* Hook. f. | *-* | **MG702537** | **MG518557** | **MG702552** | - | - | - |
| *Dicellandra descoingsii* Jacq.-Fél. | *-* | - | **MG518556** | **MG702551** | - | - | - |
| *Diplectria divaricata* Kuntze | *Clausing 236 (MJG)* | - | - | **AF215556** | **AF270746** | **AF215601** | - |
| *Dissochaeta beccariana* Cogn. | *Zhou et al. 676* (SYS) | # | # | # | # | # | MN031233 |
| *Dissochaeta gracilis* Blume | *Fan 15704* (SYS) | # | # | # | # | # | **MG644479** |
| *Dissochaeta vacillans* Blume | *Fan 15703* (SYS) | # | # | # | # | # | **MG644478** |
| *Driessenia glanduligera* Stapf | *Zhou et al. 657* (SYS) | # | # | # | # | # | MN031182 |
| *Driessenia phasmolacuna* C.W. Lin | *C.W. Lin 659* (SYS) | # | # | # | # | # | MN031199 |
| *Driessenia* sp. | *Zhou et al. 674* (SYS) | # | # | # | # | # | MN031175 |
| *Driessenia* sp*.* | *Zhou et al. 696* (SYS) | # | # | # | # | # | MN031174 |
| *Fordiophyton breviscapum* (C. Chen) Y.F. Deng & T.L. Wu | *Liu 441* (SYS) | # | # | # | # | # | **MG644455** |
| *Fordiophyton cordifolium* C.Y. Wu ex C. Chen | *Liu 430* (SYS) | # | # | # | # | # | MN031160 |
| *Fordiophyton faberi* Stapf | *Liu 480* (SYS) | # | # | # | # | # | MN031164 |
| *Fordiophyton faberi* Stapf | *Liu 588* (SYS) | # | # | # | # | # | MN031161 |
| *Fordiophyton huizhouense* S.J. Zeng & X.Y. Zhuang | *Liu 433* (SYS) | # | # | # | # | # | **MG644458** |
| *Fordiophyton jinpingense* J.H. Dai & Z.Y. Yu | *Liu 641* (SYS) | # | # | # | # | # | MN031162 |
| *Fordiophyton longipes* Y.C. Huang | *Liu 610* (SYS) | # | # | # | # | # | MN031163 |
| *Fordiophyton peperomiifolium* (Oliv.) C. Hansen | *Liu 432* (SYS) | # | # | # | # | # | **MG644459** |
| *Fordiophyton repens* Y.C. Huang ex C. Chen | *Liu 513* (SYS) | # | # | # | # | # | **MG644460** |
| *Fordiophyton strictum* Diels | *Liu 514* (SYS) | # | # | # | # | # | MN031228 |
| *Fordiophyton zhuangiae* S.J. Zeng & G.D. Tang | *Liu 574* (SYS) | # | # | # | # | # | **MG644462** |
| *Gravesia guttata* Veldkamp | *-* | **MG702540** | **MG518558** | **MG702553** | - | - | - |
| *Gravesia laxiflora* H. Perr. | *-* | - | **MG702541** | **MG702554** | - | - | - |
| *Gravesia nigrescens* (Hook.) Triana | *-* | **MG702542** | **MG518560** | **MG702555** | - | - | - |
| *Gravesia rutenbergiana* Baill. & H. Perrier | *Clausing 287 (MJG)* | - | - | **AF289370** | - | - | - |
| *Gravesia setifera* Baker | *-* | - | **MG702543** | **MG702556** | - | - | - |
| *Heteroblemma alternifolium* (Blume) Cámara-Leret, Ridd.-Num. & Veldkamp | *Clausing 184 (MJG)* | - | - | **AF289374** | - | **AF322229** | - |
| *Heteroblemma serpens* (Stapf) Cámara-Leret, Ridd.-Num. & Veldkamp | *Clausing 268 (MJG)* | - | - | **AF289376** | - | - | - |
| *Heteroblemma serpens* (Stapf) Cámara-Leret, Ridd.-Num. & Veldkamp | *Liu 671* (SYS) | # | # | # | # | # | MN031200 |
| *Kendrickia walkeri* Hook.f. | *Cultv. BG Stockholm* | - | - | **AF289371** | - | - | - |
| *Macrolenes nemorosa* (Jack) Bakh.f. | *Clausing 174 (MJG)* | - | - | **AF289372** | - | **AF289364** | - |
| *Macrolenes pachygyna* (Korth.) M.P. Nayar | *Zhou et al. 687* (SYS) | # | # | # | # | # | MN031234 |
| *Macrolenes stellulata* (Jack) Bakh.f. | *Clausing 182 (MJG)* | - | - | **AF289373** | - | - | - |
| *Medinilla amplectens* Regalado | *Zhou et al. 663* (SYS) | # | # | # | # | # | MN031219 |
| *Medinilla assamica* (C.B. Clarke) C. Chen | *Liu 590* (SYS) | # | # | # | # | # | **MG644480** |
| *Medinilla beamanii* Regalado | *Zhou et al. 658* (SYS) | # | # | # | # | # | MN031220 |
| *Medinilla chermezonii* H. Perrier | *-* | - | **MG518563** | **MG702557** | - | - | - |
| *Medinilla fengii* (S.Y. Hu) C.Y. Wu & C. Chen | *Liu 500* (SYS) | # | # | # | # | # | **MG644482** |
| *Medinilla humbertiana* H. Perrier | *Clausing 289 (MJG)* | - | - | **AF215557** | **AF215517** | **AF215602** | - |
| *Medinilla lanceata* (M.P. Nayar) C. Chen | *Liu 593* (SYS) | # | # | # | # | # | MN031232 |
| *Medinilla lophoclada* H. Perrier | *-* | - | **MG518564** | **MG702558** | - | - | - |
| *Medinilla micrantha* (Naudin) Baill. | *-* | - | **MG518565** | **MG702559** | - | - | - |
| *Medinilla petelotii* Merr. | *Liu 589* (SYS) | # | # | # | # | # | MN031218 |
| *Medinilla rubrifrons* Regalado | *Clausing 211 (MJG)* | - | - | **AF289375** | **AY456134** | **AF294838** | - |
| *Medinilla septentrionalis* (W.W. Sm.) H.L. Li | *Liu 618* (SYS) | # | # | # | # | # | MN031231 |
| *Medinilla sessiliflora* Regalado | *Clausing 154 (MJG)* | - | - | **AF289377** | - | - | - |
| *Medinilla speciosa Blume* | *Zhou et al. 669* (SYS) | # | # | # | # | # | MN031221 |
| *Medinilla squillula* Veldkamp | *-* | - | **MG518566** | **MG702560** | - | - | - |
| *Medinilla stephanostegia* Stapf | *Clausing 257 (MJG)* | - | - | **AF289378** | - | - | - |
| *Medinilla suberosa* Regalado | *Clausing 220 (MJG)* | - | - | **AF289379** | - | - | - |
| *Opisthocentra clidemioides* Hook.f. | *M.K. Caddah 578* (NY, UPCB) | # | # | # | # | # | - |
| *Oxyspora paniculata* DC. | *Liu 523* (SYS) | # | # | # | # | # | MN031212 |
| *Oxyspora teretipetiolata* (C.Y. Wu & C. Chen) W.H. Chen & Y.M. Shui | *Liu 598* (SYS) | # | # | # | # | # | **MG644476** |
| *Pachyanthus moaensis* Borhidi | Skean 4257 | - | - | **EU056128** | - | - | - |
| *Pachycentria constricta* Blume | *Clausing 263 (MJG)* | - | - | **AF289381** | - | - | - |
| *Pachycentria pulverulenta* (Jack) Clausing | *Clausing 230 (MJG)* | - | - | **AF289383** | - | - | - |
| *Phainantha laxiflora* (Triana) Gleason | *Wurdack 4190 (US)* | - | - | **JF831980** | **JF832006** | **JF832043** | - |
| *Phainantha shuariorum* C. Ulloa & D.A. Neill | *Clark 7055 (US)* | - | - | **JF831981** | **JF832007** | **JF832044** | - |
| *Phyllagathis calisaurea* C. Chen | *Liu 625* (SYS) | # | # | # | # | # | MN031188 |
| *Phyllagathis cavaleriei* (H. Lév. & Vaniot) Guillaumin | *Liu 456* (SYS) | # | # | # | # | # | **MG644422** |
| *Phyllagathis cavaleriei* var. *wilsoniana* Guillaumin | *Liu 599* (SYS) | # | # | # | # | # | **MG993330** |
| *Phyllagathis cymigera* C. Chen | *Liu 624* (SYS) | # | # | # | # | # | MN031216 |
| *Phyllagathis dispar* (Cogn.) C. Hansen | *Zhou et al. M20* (SYS) | # | # | # | # | # | **MG644429** |
| *Phyllagathis elattandra* Diels | *Liu 554* (SYS) | # | # | # | # | # | **MG644431** |
| *Phyllagathis erecta* (S.Y. Hu) C.Y. Wu ex C. Chen | *Liu 507* (SYS) | # | # | # | # | # | **MG644442** |
| *Phyllagathis fengii* C. Hansen | *Liu 520* (SYS) | # | # | # | # | # | **MG644448** |
| *Phyllagathis fordii* (Hance) C. Chen | *Liu 444* (SYS) | # | # | # | # | # | **MG644400** |
| *Phyllagathis fordii* (Hance) C. Chen var. *micrantha* C. Chen | *Liu 580* (SYS) | # | # | # | # | # | **MG644401** |
| *Phyllagathis gigantifolia* M.P. Nayar | *Zhou et al. 659* (SYS) | # | # | # | # | # | MN031177 |
| *Phyllagathis gracilis* (Hand.-Mazz.) C. Chen | *Liu 457* (SYS) | # | # | # | # | # | **MG644403** |
| *Phyllagathis guidongensis* K.M. Liu & J. Tian | *Liu 472* (SYS) | # | # | # | # | # | **MG644404** |
| *Phyllagathis gymnantha* Korth. | *C.W. Lin 625* (TAIF) | # | # | # | # | # | **MG993334** |
| *Phyllagathis hispida* King | *Zhou et al. M49* (SYS) | # | # | # | # | # | **MG644435** |
| *Phyllagathis hispidissima* (C. Chen) C. Chen | *Liu 604* (SYS) | # | # | # | # | # | MN031187 |
| *Phyllagathis latisepala* C. Chen | *Liu 557* (SYS) | # | # | # | # | # | **MG644405** |
| *Phyllagathis lii* C.W. Lin, Chien F. Chen & T.Y.A. Yang | *C.W. Lin 667* (TAIF) | # | # | # | # | # | MN031180 |
| *Phyllagathis longearistata* C. Chen | *Liu 498* (SYS) | # | # | # | # | # | **MG644406** |
| *Phyllagathis longicalcarata* C. Hansen | *Liu 640* (SYS) | # | # | # | # | # | MN031236 |
| *Phyllagathis longicalcarata* C. Hansen | *Liu 721* (SYS) | # | # | # | # | # | MN031166 |
| *Phyllagathis longiradiosa* (C. Chen) C. Chen | *Liu 486* (SYS) | # | # | # | # | # | MN031235 |
| *Phyllagathis longiradiosa* var. *pulchella* C. Chen | *Liu 485* (SYS) | # | # | # | # | # | **MG644409** |
| *Phyllagathis melastomatoides* (Merr. & Chun) W.C. Ko | *Liu 447* (SYS) | # | # | # | # | # | **MG644424** |
| *Phyllagathis millelunata* C.W. Lin, Chien F. Chen & T.Y.A. Yang | *C.W. Lin 582* (TAIF) | # | # | # | # | # | **MG993335** |
| *Phyllagathis nudipes* C. Chen | *Liu 435* (SYS) | # | # | # | # | # | **MG644394** |
| *Phyllagathis oligotricha* Merr. | *Liu 468* (SYS) | # | # | # | # | # | **MG644395** |
| *Phyllagathis osmantha* (M.P. Nayar) Cellin. | *C.W. Lin 567* (TAIF) | # | # | # | # | # | **MG993336** |
| *Phyllagathis ovalifolia* H.L. Li | *Liu 512* (SYS) | # | # | # | # | # | **MG644425** |
| *Phyllagathis plagiopetala* C. Chen | *Liu 459* (SYS) | # | # | # | # | # | **MG644410** |
| *Phyllagathis plagiopetala* C. Chen | *Liu 460* (SYS) | # | # | # | # | # | **MG644411** |
| *Phyllagathis postrata* C. Hansen | *C.W. Lin 640* (TAIF) | # | # | # | # | # | **MG993332** |
| *Phyllagathis rajah* C.W. Lin, Chien F. Chen & T.Y.A. Yang | *C.W. Lin 644* (TAIF) | # | # | # | # | # | **MG993339** |
| *Phyllagathis rotundifolia* (Jack) Blume | *Zhou et al. M50* (SYS) | # | # | # | # | # | **MG644436** |
| *Phyllagathis rufa* (Stapf) Cellin. | *Zhou et al. 679* (SYS) | # | # | # | # | # | MN031209 |
| *Phyllagathis scortechinii* King | *Zhou et al. M48* (SYS) | # | # | # | # | # | **MG644437** |
| *Phyllagathis sessilifolia* C. Hansen | *Q. Fan 17311* (SYS) | # | # | # | # | # | MN031217 |
| *Phyllagathis setotheca* var. *setotuba* C. Chen | *Liu 576* (SYS) | # | # | # | # | # | **MG644426** |
| *Phyllagathis* sp. nov*.* | *C.W. Lin 668* (TAIF) | # | # | # | # | # | MN031169 |
| *Phyllagathis stellata* C.W. Lin & C.H. Lee | *C.W. Lin 643* (TAIF) | # | # | # | # | # | **MG993333** |
| *Phyllagathis stenophylla* (Merr. & Chun) H.L. Li | *Liu 453* (SYS) | # | # | # | # | # | **MG644427** |
| *Phyllagathis suberalata* C. Hansen | *Q. Fan 17316* (SYS) | # | # | # | # | # | MN031238 |
| *Phyllagathis suberalata* C. Hansen | *Q. Fan 17327* (SYS) | # | # | # | # | # | MN031222 |
| *Phyllagathis tentaculifera* C. Hansen | *Liu 722* (SYS) | # | # | # | # | # | MN031171 |
| *Phyllagathis tentaculifera* C. Hansen | *Liu 723* (SYS) | # | # | # | # | # | MN031172 |
| *Phyllagathis tetrandra* Diels | *Liu 519* (SYS) | # | # | # | # | # | **MG644432** |
| *Phyllagathis velutina* (Diels) C. Chen | *Liu 509* (SYS) | # | # | # | # | # | **MG644413** |
| *Phyllagathis wallacei* C.W. Lin, Chien F. Chen & T.Y.A. Yang | *Zhou et al. 686* (SYS) | # | # | # | # | # | MN031198 |
| *Phyllagathis xinyiensis* Z.J. Feng | *Liu 582* (SYS) | # | # | # | # | # | **MG644428** |
| *Plagiopetalum esquirolii* (H. Lév.) Rehder | *Liu 594* (SYS) | # | # | # | # | # | MN031202 |
| *Plagiopetalum serratum* (Diels) Diels | *Liu 717* (SYS) | # | # | # | # | # | MN031170 |
| *Plethiandra cuneata* Stapf | *Clausing 129 (MJG)* | - | - | AF289384 | - | - | - |
| *Plethiandra hookeri* Stapf | *Clausing 219 (MJG)* | - | - | AF289385 | - | - | - |
| *Sarcopyramis bodinieri* H. Lév. | *Liu 502* (SYS) | # | # | # | # | # | MN031206 |
| *Sarcopyramis nepalensis* Wall. | *Liu 581* (SYS) | # | # | # | # | # | MN031208 |
| *Sarcopyramis nepalensis* Wall. | *Liu 628* (SYS) | # | # | # | # | # | MN031207 |
| *Scorpiothyrsus oligotrichus* H.L. Li | *Liu 454* (SYS) | # | # | # | # | # | **MG644440** |
| *Scorpiothyrsus shangszeensis* C. Chen | *Liu 626* (SYS) | # | # | # | # | # | MN031186 |
| *Sonerila borneensis* Cogn. | *Zhou et al. 684* (SYS) | # | # | # | # | # | MN031190 |
| *Sonerila cantonensis* Stapf | *Liu 510* (SYS) | # | # | # | # | # | MN031194 |
| *Sonerila cantonensis* Stapf | *Liu 449* (SYS) | # | # | # | # | # | **MG644491** |
| *Sonerila parviflora* Cogn. | *Zhou et al. 700* (SYS) | # | # | # | # | # | MN031223 |
| *Sonerila plagiocardia* Diels | *Liu 443* (SYS) | # | # | # | # | # | **MG644498** |
| *Sonerila plagiocardia* Diels | *Liu 642* (SYS) | # | # | # | # | # | MN031191 |
| *Sonerila pulchella* Stapf | *Zhou et al. 668* (SYS) | # | # | # | # | # | MN031193 |
| *Sonerila velutina* Cogn. | *Zhou et al. 683* (SYS) | # | # | # | # | # | MN031224 |
| *Sonerila yunnanensis* Jeffrey ex W.W. Sm. | *Liu 621* (SYS) | # | # | # | # | # | MN031192 |
| *Sporoxeia clavicalcarata* C. Chen | *Liu 716* (SYS) | # | # | # | # | # | MN031176 |
| *Sporoxeia latifolia* (H.L. Li) C.Y. Wu & Y.C. Huang | *Liu 524* (SYS) | # | # | # | # | # | MN031201 |
| *Sporoxeia petelotii* (Merr.) C. Hansen | *Liu 719* (SYS) | # | # | # | # | # | MN031173 |
| *Styrophyton caudatum* (Diels) S.Y. Hu | *Liu 615* (SYS) | # | # | # | # | # | MN031203 |
| *Tashiroea* sp. nov*.* | *Liu 476* (SYS) | # | # | # | # | # | MN031204 |
| *Tashiroea* sp. nov*.* | *Liu 568* (SYS) | # | # | # | # | # | MN031214 |
| *Tigridiopalma magnifica* C. Chen | *Liu 429* (SYS) | # | # | # | # | # | **MG644449** |
