# Supplementary material for: Analyses of Plastome Sequences Improve Phylogenetic Resolution and Provide New Insight Into the Evolutionary History of Asian Sonerileae/Dissochaeteae
Source: Front Plant Sci. 2019 Nov 21;10:1477. doi: 10.3389/fpls.2019.01477 (PMC6881482; doi:10.3389/fpls.2019.01477)
Supplement: Supplementary file 8 [file Table_3.docx]

Table S3. Summary of model selection for five partitioning schemes and models used in ML/BI analysis.

| Number of partition | dataset | Number of sites | Best fit model | ML | BI |
| --- | --- | --- | --- | --- | --- |
| 1 | The Melastomataceae dataset | 129481 | TVM+I+G | GTR+G | GTR+G/  GTR+I+G |
| 2 | Coding region | 77002 | GTR+I+G | GTR+G | GTR+G/  GTR+I+G |
|  | Noncoding region | 55583 | TVM +I+G | GTR+G | GTR+G/  GTR+I+G |
| 3 | LSC | 85815 | GTR+I+G | GTR+G | GTR+G/  GTR+I+G |
|  | IR | 26500 | GTR+I+G | GTR+G | GTR+G/  GTR+I+G |
|  | SSC | 16897 | TVM+I+G | GTR+G | GTR+G/  GTR+I+G |
| 6 | Codon1 | 23407 | GTR+I+G | GTR+G | GTR+G/  GTR+I+G |
|  | Codon2 | 23407 | GTR+I+G | GTR+G | GTR+G/  GTR+I+G |
|  | Codon3 | 23407 | GTR+I+G | GTR+G | GTR+G/  GTR+I+G |
|  | Noncoding region | 55583 | TVM+I+G | GTR+G | GTR+G/  GTR+I+G |
|  | tRNAs | 2283 | TVM+I+G | GTR+G | GTR+G/  GTR+I+G |
|  | rRNAs | 4528 | GTR+I+G | GTR+G | GTR+G/  GTR+I+G |
| 15* | Subset1 | 7275 | GTR+I+G | GTR+G | GTR+I+G |
|  | Subset2 | 11307 | GTR+I+G | GTR+G | GTR+I+G |
|  | Subset3 | 2321 | GTR+I+G | GTR+G | GTR+I+G |
|  | Subset4 | 969 | GTR+G | GTR+G | GTR+G |
|  | Subse5 | 9711 | GTR+I+G | GTR+G | GTR+I+G |
|  | Subset6 | 4968 | GTR+I+G | GTR+G | GTR+I+G |
|  | Subset7 | 952 | GTR+I+G | GTR+G | GTR+I+G |
|  | Subset8 | 3555 | GTR+I+G | GTR+G | GTR+I+G |
|  | Subset9 | 10683 | GTR+I+G | GTR+G | GTR+I+G |
|  | Subset10 | 5187 | GTR+G | GTR+G | GTR+G |
|  | Subset11 | 6315 | GTR+I+G | GTR+G | GTR+I+G |
|  | Subset12 | 6978 | GTR+I+G | GTR+G | GTR+I+G |
|  | Subset13 | 55583 | GTR+I+G | GTR+G | GTR+I+G |
|  | Subset14 | 2283 | GTR+I+G | GTR+G | GTR+I+G |
|  | Subset15 | 4528 | GTR+I+G | GTR+G | GTR+I+G |

* Subset1: atp_2, atp_3, atp_1, clpP_matK_1, clpP_matK_2, clpP_matK_3

Subset2: cemA_2, cemA_3, cemA_1, ndh_3, ndh_1, ndh_2

Subset3: accD_1, rbcL_2, accD_3, accD_2

Subset4: ccsA_3, ccsA_2, ccsA_1

Subset5: psb_2, psb_3, psb_1, pet_2, pet_1, pet_3, ycf3_1, ycf3_3, ycf3_2

Subset6: psa_3, psa_2, psa_1

Subset7: rbcL_1, rbcL_3

Subset8: ycf4_3, ycf4_2, ycf4_1, rpl_3, rpl_1, rpl_2

Subset9: rpo_1, rpo_3, rpo_2

Subset10: rps_1, rps_2, rps_3

Subset11: ycf1_1, ycf1_2, ycf1_3

Subset12: ycf2_2, ycf2_1, ycf2_3

Subset13: noncoding

Subset14: tRNA

Subset15: rRNA
