# Supplementary material for: Analyses of Plastome Sequences Improve Phylogenetic Resolution and Provide New Insight Into the Evolutionary History of Asian Sonerileae/Dissochaeteae
Source: Front Plant Sci. 2019 Nov 21;10:1477. doi: 10.3389/fpls.2019.01477 (PMC6881482; doi:10.3389/fpls.2019.01477)
Supplement: Supplementary file 9 [file Table_4.docx]

**Table. S4.** Species sampled in this study were coded as present (1) or absent (0) for each of the following five areas: A. north America; B. south America; C. Indo-Burma, also including part of southern and western Yunnan, southernmost Guangxi and Guangdong, and Hainan Island; D. Sundaland; E. Sino-Japanese region, including most of central and southern mainland China, Taiwan, and Ryukyu.

| Species | A | B | C | D | E |
| --- | --- | --- | --- | --- | --- |
| *Allomaieta villosa* | 0 | 1 | 0 | 0 | 0 |
| *Allomorphia balansae* | 0 | 0 | 1 | 0 | 0 |
| *Allomorphia* sp. | 0 | 0 | 0 | 1 | 0 |
| *Allomorphia urophylla* 592 | 0 | 0 | 1 | 0 | 0 |
| *Allomorphia urophylla* 620 | 0 | 0 | 1 | 0 | 0 |
| *Allomorphia urophylla* 718 | 0 | 0 | 1 | 0 | 0 |
| *Anerincleistus bracteatus* | 0 | 0 | 0 | 1 | 0 |
| *Anerincleistus bullatus* | 0 | 0 | 0 | 1 | 0 |
| *Anerincleistus macrophyllus* | 0 | 0 | 0 | 1 | 0 |
| *Anerincleistus phyllagathoides* | 0 | 0 | 0 | 1 | 0 |
| *Anerincleistus quintuplinervis* | 0 | 0 | 0 | 1 | 0 |
| *Anerincleistus sertuliferus* | 0 | 0 | 0 | 1 | 0 |
| *Anerincleistus setulosus* | 0 | 0 | 0 | 1 | 0 |
| *Anerincleistus* sp. | 0 | 0 | 0 | 1 | 0 |
| *Astronia smilacifolia* | 0 | 0 | 0 | 1 | 0 |
| *Barthea barthei* | 0 | 0 | 1 | 0 | 1 |
| *Bertolonia acuminata* | 0 | 1 | 0 | 0 | 0 |
| *Blakea schlimii* | 0 | 1 | 0 | 0 | 0 |
| *Blastus auriculatus* | 0 | 0 | 1 | 0 | 0 |
| *Blastus cavaleriei* | 0 | 0 | 0 | 0 | 1 |
| *Blastus cochinchinensis* | 0 | 0 | 1 | 0 | 1 |
| *Blastus dunnianus* | 0 | 0 | 0 | 0 | 1 |
| *Blastus ernae* | 0 | 0 | 0 | 0 | 1 |
| *Blastus mollissimus* | 0 | 0 | 1 | 0 | 1 |
| *Bredia amoena* 547 | 0 | 0 | 0 | 0 | 1 |
| *Bredia amoena* 571 | 0 | 0 | 0 | 0 | 1 |
| *Bredia biglandularis* | 0 | 0 | 1 | 0 | 0 |
| *Bredia changii* | 0 | 0 | 0 | 0 | 1 |
| *Bredia dulanica* | 0 | 0 | 0 | 0 | 1 |
| *Bredia esquirolii* | 0 | 0 | 0 | 0 | 1 |
| *Bredia gibba* | 0 | 0 | 0 | 0 | 1 |
| *Bredia hirsuta* 563 | 0 | 0 | 0 | 0 | 1 |
| *Bredia hirsuta* 632 | 0 | 0 | 0 | 0 | 1 |
| *Bredia hirsuta* 634 | 0 | 0 | 0 | 0 | 1 |
| *Bredia hirsuta* var. *scandens* | 0 | 0 | 0 | 0 | 1 |
| *Bredia longiloba* | 0 | 0 | 0 | 0 | 1 |
| *Bredia microphylla* | 0 | 0 | 0 | 0 | 1 |
| *Bredia okinawensis*(=*Tashiroea okinawensis*) | 0 | 0 | 0 | 0 | 1 |
| *Bredia oldhamii* | 0 | 0 | 0 | 0 | 1 |
| *Bredia quadrangularis* | 0 | 0 | 0 | 0 | 1 |
| *Bredia repens* | 0 | 0 | 0 | 0 | 1 |
| *Bredia rotundifolia* | 0 | 0 | 0 | 0 | 1 |
| *Bredia sessilifolia* | 0 | 0 | 1 | 0 | 1 |
| *Bredia sinensis*(=*Tashiroea sinensis*) | 0 | 0 | 0 | 0 | 1 |
| *Bredia* sp*.* | 0 | 0 | 1 | 0 | 0 |
| *Bredia tuberculate* 579 | 0 | 0 | 0 | 0 | 1 |
| *Bredia tuberculate* 629 | 0 | 0 | 0 | 0 | 1 |
| *Bredia yaeyamensis*(=*Tashiroea yaeyamensis*) | 0 | 0 | 0 | 0 | 1 |
| *Bredia yunnanensis* | 0 | 0 | 0 | 0 | 1 |
| *Cyphotheca montana* | 0 | 0 | 1 | 0 | 0 |
| *Dissochaeta beccariana* | 0 | 0 | 0 | 1 | 0 |
| *Dissochaeta gracilis* | 0 | 0 | 0 | 1 | 0 |
| *Dissochaeta vacillans* | 0 | 0 | 0 | 1 | 0 |
| *Driessenia glanduligera* | 0 | 0 | 0 | 1 | 0 |
| *Driessenia phasmolacuna* | 0 | 0 | 0 | 1 | 0 |
| *Driessenia* sp*.* 674 | 0 | 0 | 0 | 1 | 0 |
| *Driessenia* sp*.* 696 | 0 | 0 | 0 | 1 | 0 |
| *Eriocnema fulva* | 0 | 1 | 0 | 0 | 0 |
| *Fordiophyton breviscapum* | 0 | 0 | 0 | 0 | 1 |
| *Fordiophyton cordifolium* | 0 | 0 | 0 | 0 | 1 |
| *Fordiophyton faberi* 480 | 0 | 0 | 0 | 0 | 1 |
| *Fordiophyton faberi* 588 | 0 | 0 | 0 | 0 | 1 |
| *Fordiophyton huizhouense* | 0 | 0 | 0 | 0 | 1 |
| *Fordiophyton jinpingense* | 0 | 0 | 1 | 0 | 0 |
| *Fordiophyton longipes* | 0 | 0 | 1 | 0 | 0 |
| *Fordiophyton peperomiifolium* | 0 | 0 | 0 | 0 | 1 |
| *Fordiophyton repens* | 0 | 0 | 1 | 0 | 0 |
| *Fordiophyton strictum* | 0 | 0 | 1 | 0 | 0 |
| *Fordiophyton zhuangiae* | 0 | 0 | 1 | 0 | 0 |
| *Graffenrieda moritziana* | 0 | 1 | 0 | 0 | 0 |
| *Henriettea barkeri* | 0 | 1 | 0 | 0 | 0 |
| *Heteroblemma serpens* | 0 | 0 | 0 | 1 | 0 |
| *Macrolenes pachygyna* | 0 | 0 | 0 | 1 | 0 |
| *Medinilla amplectens* | 0 | 0 | 0 | 1 | 0 |
| *Medinilla assamica* | 0 | 0 | 1 | 0 | 0 |
| *Medinilla beamanii* | 0 | 0 | 0 | 1 | 0 |
| *Medinilla fengii* | 0 | 0 | 1 | 0 | 1 |
| *Medinilla lanceata* | 0 | 0 | 1 | 0 | 0 |
| *Medinilla petelotii* | 0 | 0 | 1 | 0 | 1 |
| *Medinilla septentrionalis* | 0 | 0 | 1 | 0 | 1 |
| *Medinilla speciosa* | 0 | 0 | 0 | 1 | 0 |
| *Melastoma candidum* | 0 | 0 | 1 | 0 | 1 |
| *Memecylon ligustrifolium* | 0 | 0 | 1 | 0 | 1 |
| *Merianthera pulchra* | 0 | 1 | 0 | 0 | 0 |
| *Miconia dodecandra* | 0 | 1 | 0 | 0 | 0 |
| *Nepsera aquatica* | 0 | 1 | 0 | 0 | 0 |
| *Ochthocharis bornensis* | 0 | 0 | 0 | 1 | 0 |
| *Opisthocentra clidemioides* | 0 | 1 | 0 | 0 | 0 |
| *Oxyspora paniculata* | 0 | 0 | 1 | 0 | 1 |
| *Oxyspora teretipetiolata* | 0 | 0 | 1 | 0 | 0 |
| *Phyllagathis calisaurea* | 0 | 0 | 1 | 0 | 0 |
| *Phyllagathis cavaleriei* | 0 | 0 | 1 | 0 | 1 |
| *Phyllagathis cavaleriei* var. *wilsoniana* | 0 | 0 | 0 | 0 | 1 |
| *Phyllagathis cymigera* | 0 | 0 | 1 | 0 | 0 |
| *Phyllagathis dispar* | 0 | 0 | 0 | 1 | 0 |
| *Phyllagathis elattandra* | 0 | 0 | 1 | 0 | 1 |
| *Phyllagathis erecta* | 0 | 0 | 1 | 0 | 0 |
| *Phyllagathis fengii* | 0 | 0 | 1 | 0 | 0 |
| *Phyllagathis fordii* | 0 | 0 | 1 | 0 | 1 |
| *Phyllagathis fordii* var. *micrantha* | 0 | 0 | 0 | 0 | 1 |
| *Phyllagathis gigantifolia* | 0 | 0 | 0 | 1 | 0 |
| *Phyllagathis gracilis* | 0 | 0 | 0 | 0 | 1 |
| *Phyllagathis guidongensis* | 0 | 0 | 0 | 0 | 1 |
| *Phyllagathis gymnantha* | 0 | 0 | 0 | 1 | 0 |
| *Phyllagathis hispida* | 0 | 0 | 0 | 1 | 0 |
| *Phyllagathis hispidissima* 604 | 0 | 0 | 1 | 0 | 0 |
| *Phyllagathis hispidissima* 640 | 0 | 0 | 1 | 0 | 0 |
| *Phyllagathis hispidissima* 721 | 0 | 0 | 1 | 0 | 0 |
| *Phyllagathis latisepala* | 0 | 0 | 0 | 0 | 1 |
| *Phyllagathis lii* | 0 | 0 | 0 | 1 | 0 |
| *Phyllagathis longearistata* | 0 | 0 | 0 | 0 | 1 |
| *Phyllagathis longiradiosa* | 0 | 0 | 1 | 0 | 0 |
| *Phyllagathis longiradiosa* var. *pulchella* | 0 | 0 | 1 | 0 | 0 |
| *Phyllagathis melastomatoides* | 0 | 0 | 1 | 0 | 0 |
| *Phyllagathis millelunata* | 0 | 0 | 0 | 1 | 0 |
| *Phyllagathis nudipes* | 0 | 0 | 0 | 0 | 1 |
| *Phyllagathis oligotricha* | 0 | 0 | 0 | 0 | 1 |
| *Phyllagathis osmantha* | 0 | 0 | 0 | 1 | 0 |
| *Phyllagathis ovalifolia* | 0 | 0 | 1 | 0 | 0 |
| *Phyllagathis plagiopetala* 459 | 0 | 0 | 0 | 0 | 1 |
| *Phyllagathis plagiopetala* 460 | 0 | 0 | 0 | 0 | 1 |
| *Phyllagathis postrata* | 0 | 0 | 1 | 0 | 0 |
| *Phyllagathis rajah* | 0 | 0 | 0 | 1 | 0 |
| *Phyllagathis rotundifolia* | 0 | 0 | 0 | 1 | 0 |
| *Phyllagathis rufa* | 0 | 0 | 0 | 1 | 0 |
| *Phyllagathis scortechinii* | 0 | 0 | 0 | 1 | 0 |
| *Phyllagathis sessilifolia* | 0 | 0 | 1 | 0 | 0 |
| *Phyllagathis setotheca* var. *setotuba* | 0 | 0 | 1 | 0 | 0 |
| *Phyllagathis* sp. nov. (Lin 668) | 0 | 0 | 0 | 1 | 0 |
| *Phyllagathis stellata* | 0 | 0 | 0 | 1 | 0 |
| *Phyllagathis stenophylla* | 0 | 0 | 1 | 0 | 0 |
| *Phyllagathis suberalata* (Fan 17316) | 0 | 0 | 1 | 0 | 0 |
| *Phyllagathis suberalata* (Fan 17327) | 0 | 0 | 1 | 0 | 0 |
| *Phyllagathis tentaculifera* 722 | 0 | 0 | 1 | 0 | 0 |
| *Phyllagathis tentaculifera* 723 | 0 | 0 | 1 | 0 | 0 |
| *Phyllagathis tetrandra* | 0 | 0 | 1 | 0 | 0 |
| *Phyllagathis velutina* | 0 | 0 | 1 | 0 | 0 |
| *Phyllagathis wallacei* | 0 | 0 | 0 | 1 | 0 |
| *Phyllagathis xinyiensis* | 0 | 0 | 1 | 0 | 0 |
| *Plagiopetalum esquirolii* | 0 | 0 | 1 | 0 | 1 |
| *Plagiopetalum serratum* | 0 | 0 | 1 | 0 | 0 |
| *Pternandra korthalsiana* | 0 | 0 | 0 | 1 | 0 |
| *Pternandra tesellata* | 0 | 0 | 0 | 1 | 0 |
| *Pterogastra divaricata* | 0 | 1 | 0 | 0 | 0 |
| *Rhexia virginica* | 1 | 0 | 0 | 0 | 0 |
| *Rhynchanthera bracteata* | 0 | 1 | 0 | 0 | 0 |
| *Salpinga maranonensis* | 0 | 1 | 0 | 0 | 0 |
| *Sarcopyramis bodinieri* | 0 | 0 | 1 | 0 | 1 |
| *Sarcopyramis napalensis* 581 | 0 | 0 | 1 | 0 | 1 |
| *Sarcopyramis napalensis* 628 | 0 | 0 | 1 | 0 | 1 |
| *Scorpiothyrsus oligotrichus* | 0 | 0 | 1 | 0 | 0 |
| *Scorpiothyrsus shangszeensis* | 0 | 0 | 1 | 0 | 0 |
| *Sonerila borneensis* | 0 | 0 | 0 | 1 | 0 |
| *Sonerila cantonensis* 449 | 0 | 0 | 1 | 0 | 1 |
| *Sonerila cantonensis* 510 | 0 | 0 | 1 | 0 | 1 |
| *Sonerila parviflora* | 0 | 0 | 0 | 1 | 0 |
| *Sonerila plagiocardia* 443 | 0 | 0 | 1 | 0 | 1 |
| *Sonerila plagiocardia* 642 | 0 | 0 | 1 | 0 | 1 |
| *Sonerila pulchella* | 0 | 0 | 0 | 1 | 0 |
| *Sonerila velutina* | 0 | 0 | 0 | 1 | 0 |
| *Sonerila yunnanensis* | 0 | 0 | 1 | 0 | 0 |
| *Sporoxeia clavicalcarata* | 0 | 0 | 1 | 0 | 0 |
| *Sporoxeia latifolia* | 0 | 0 | 1 | 0 | 1 |
| *Sporoxeia petelotii* | 0 | 0 | 1 | 0 | 0 |
| *Styrophyton caudatum* | 0 | 0 | 1 | 0 | 0 |
| *Tashiroea* sp. nov. 476 | 0 | 0 | 1 | 0 | 0 |
| *Tashiroea* sp. nov. 568 | 0 | 0 | 0 | 0 | 1 |
| *Tibouchina longifolia* | 0 | 1 | 0 | 0 | 0 |
| *Tigridiopalma magnifica* | 0 | 0 | 1 | 0 | 1 |
| *Triolena amazonica* | 0 | 1 | 0 | 0 | 0 |
