# Supplementary material for: Analyses of Plastome Sequences Improve Phylogenetic Resolution and Provide New Insight Into the Evolutionary History of Asian Sonerileae/Dissochaeteae
Source: Front Plant Sci. 2019 Nov 21;10:1477. doi: 10.3389/fpls.2019.01477 (PMC6881482; doi:10.3389/fpls.2019.01477)
Supplement: Supplementary file 10 [file Table_5.docx]

**Table S5.** Potential diagnostic characters and geographical distribution of the 15 clades recovered in analyses of nrITS and chloroplast genome.

| clade | Habit | Leaf | Inflorescence | Stamen | Fruit | Distribution |
| --- | --- | --- | --- | --- | --- | --- |
| *Dissochaeta* | Woody climbers | Ovate, elliptic, or lanceolate | Terminal or axillary, cymose | Stamens 8, dimorphic | Berry | Indo-Burma and Malesia |
| *Pseudodissochaeta* | Shrubs, rarely trees erect or scrambling | Ovate, elliptic, or lanceolate | Terminal or axillary, cymose or cymose paniculate | Stamens 8, isomorphic | Berry | Indo-Burma |
| *Tashiroea* | Shrublets or shrubs | Stiffly papery to subleathery, glabrous; | Terminal, cymose panicles | Stamen 8, isomorphic or dimorphic | Capsule, ovary crown evanescent, rarely enlarged; placental column not 4-horned, placenta non-thready | Southeastern China, Taiwan, Ryukyu Islands |
| *Bredia* | Herbs, shrublets, or shrubs | More or less ovate, papery, pubescent | Terminal, umbels or cymose panicles | Stamen 8, isomorphic or dimorphic | Capsule, ovary crown enlarged and persistent; placental column not 4-horned, placenta non-thready | Central and southern China, Taiwan, Ryukyu Islands, northern Vietnam |
| Unnamed clade 1 | Shrubs, rarely caulescent herbs | Ovate, elliptic to oblong-lanceolate, papery | Terminal, umbellate or cymose, rarely solitary flower, terminal or axillary | Stamen 8, isomorphic, connectives dorsally spurred | Capsule, ovary crown enlarged, placental column horned, placenta thready | Southernmost China, Vietnam, and Borneo |
| *Phyllagathis elattandra-P. terandra* | Herbs | Elliptic to suborbicular, papery | Terminal, umbellate | Stamens 4, or 8 but with  the inner whorl sterile | Capsule, 4-sided, ovary crown enlarged and persistent; placental column not 4-horned, placenta non-thready | Southernmost China |
| *Anerincleistus* | Shrubs or herbs | Leaves subequal or unequal, orbicular to lanceolate | Terminal or axillary, umbellate or cymose panicles | Stamen 8, isomorphic | Capsule, ovary crown often enlarged, placenta column horned or not, placenta thready or not | Malay peninsula, Sumatra, Borneo |
| *Cyphotheca* | Shrubs | Ovate or ovate-oblong, papery | Terminal, cymose umbellate or corymbose | Stamen 8, dimorphic or isomorphic, connectives thickened | Capsule, ovary crown enlarged, placental column horned, placenta thready | Southeastern Yunnan, China |
| *Scorpiothysus* | Shrubs | Papery, ovate or elliptic, veins 3 or 4 on each side of the midvein, margin denticulate | Terminal, scorpioid cymose panicles, cymose or cymose panicles | Stamen 8, isomorphic, connective dorsally shortly spurred | Capsule, cup-shaped, crown enlarged or not, placental column horned or not, placenta thready or not | Southernmost China |
| *Sporoxeia* | Shrubs | Papery, elliptic, ovate to orbicular | Axillary or terminal, umbellate or cymes | Stamens 8, isomorphic, connective dorsally spurred | Capsule, crown enlarged, placental column horned, placenta thready or not | Southern and western Yunnan, China, Vietnam |
| *Styrophyton* | Shrubs | Papery, lanceolate-elliptic to broadly ovate | Terminal, cymose panicle or spicate with sessile small flowers | Stamens 8, isomorphic | Capsule, ovary crown absent, placental column not horned, placenta not thready | Southernmost China, northern Vietnam |
| *Fordiophyton* | Herbs | Ovate-lanceolate to ovate-orbicular, papery, rarely subleathery | Terminal, umbellate or cymose panicles | Stamens 8, distinctly dimorphic, connective often minutely inflated, not tuberculate or spurred | Capsule, not exceeding hypanthium, placental column not horned, placenta not thready | Central and southern China, Vietnam |
| *Medinilla* | Shrubs, epiphytic | Obovate to elliptic, 3-, 5-, or 7-nerved, glabrous | Terminal or axillary, cymes, corymb-panicles, cymose panicles, or glomerulate | Stamens 8, isomorphic | Berry | Southeast Asia |
| *Sonerila* | Herbs, shrublets | Often oblique | Terminal or axillary, scorpioid cymes or subumbellate, pedunculate | Stamens 3 or 6, connective not inflated | Capsule, crown exserted from hypanthium, placental column not horned, placenta not thready | Southeast Asia |
| *Sarcopyramis* | Herbs | Ovate to elliptic | Terminal cymes, 3–5-flowered | Stamens 8, small, isomorphic | Capsule cup-shaped, crown membranous, calyx lobes apex often pubescent or with a fimbriate membranous disk | Indo-Burma and Malesia |
